# On-Surface Synthesis of a Radical 2D Supramolecular Organic Framework

**DOI:** 10.1021/jacs.3c13702

**Published:** 2024-01-25

**Authors:** Federico Frezza, Adam Matěj, Ana Sánchez-Grande, Manuel Carrera, Pingo Mutombo, Manish Kumar, David Curiel, Pavel Jelínek

**Affiliations:** † Institute of Physics of Czech Academy of Sciences, Cukrovarnická 10, 16200 Prague 6 ,Czech Republic; ‡ Faculty of Nuclear Sciences and Physical Engineering, Czech Technical University in Prague, Břehová 78/7,11519 Prague 1, Czech Republic; § Department of Physical Chemistry, Faculty of Science, Palacký University, 17. Listopadu 12, 779 00 Olomouc, Czech Republic; ∥ Department of Organic Chemistry, University of Murcia, Campus of Espinardo, 30100 Murcia, Spain; ⊥ Département de Raffinage et Pétrochimie, Faculté de Pétrole, Gaz et Énergies Renouvelables, Université de Kinshasa, BP 127 Kinshasa XI, République Démocratique du Congo; # CATRIN-RCPTM, Palacký University, Šlechtitelu° 27, 783 71 Olomouc, Czech Republic

## Abstract

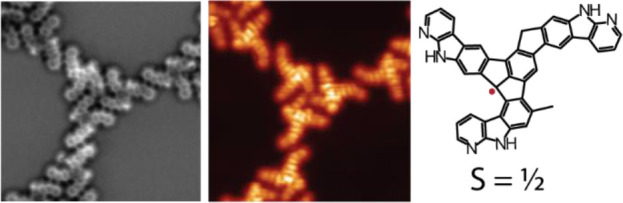

The design of supramolecular organic radical cages and frameworks is one of the main challenges in supramolecular chemistry. Their interesting material properties and wide applications make them very promising for (photo)redox catalysis, sensors, or host–guest spin–spin interactions. However, the high reactivity of radical organic systems makes the design of such supramolecular radical assemblies challenging. Here, we report the on-surface synthesis of a purely organic supramolecular radical framework on Au(111), by combining supramolecular and on-surface chemistry. We employ a tripodal precursor, functionalized with 7-azaindole groups that, catalyzed by a single gold atom on the surface, forms a radical molecular product constituted by a π-extended fluoradene-based radical core. The radical products self-assemble through hydrogen bonding, leading to extended 2D domains ordered in a Kagome-honeycomb lattice. This approach demonstrates the potential of on-surface synthesis for developing 2D supramolecular radical organic chemistry.

## Introduction

Large-scale fabrication of ordered structures with atomic precision is one of the main goals of nanotechnology. Different bottom-up approaches have been employed to this aim, either via physical or chemical forces of organic and/or inorganic compounds.^1^ Remarkably, one of the most successful strategies for the growth of large-scale defect-free ordered structures is the design of supramolecular frameworks, which are stabilized by noncovalent intermolecular interactions.^2,3^ Typically, the molecular units can be rationally designed to incorporate functional groups that control the noncovalent interactions within the self-assembled system, thus spontaneously inducing the formation of ordered supramolecular organic frameworks. The main advantage of this strategy is that, in contrast to 2D-covalent organic frameworks (2D-COFs),^4^ no covalent bond-forming reaction is required for the network growth, but the material is self-organized, and the dynamic assembly can contribute to the reduction of structural defects in large domains.

The control of the molecular arrangement on surfaces has gained much attention during the past few years due to the large variety of applications of two-dimensional (2D) surface-confined supramolecular nanostructures.^5−9^ Different supramolecular assemblies have been reported, where the role of the surface, the coverage-dependence, and kinetic or thermodynamic control allow tailoring the 2D nanostructures.^10,11^ Furthermore, supramolecular bicomponent frameworks have also been explored, based on purely organic compounds^12,13^ or inorganic–organic bicomponents.^14−16^ Countless 2D nanostructures have been reported to date, with tunable size and reactivity of the nanopores, giving rise to the formation of host–guest systems with high specificity.^17,18^ In this regard, hydrogen bonding becomes a particularly useful tool, considering the directionality, high energy (within the context of noncovalent interactions), and variety of functional groups and structures that can set this interaction.^19^

One of the main challenges in supramolecular chemistry is the synergy of the nanopatterning field and radical organic chemistry, known as supramolecular radical chemistry, which consists of the ordered arrangement of organic radicals systems by noncovalent interactions.^20−23^ Long-range supramolecular radical frameworks have potential applications in quantum technology, sensors, and photoredox catalysis.^24−26^ Nevertheless, the high reactivity of organic radicals makes their incorporation into the host network difficult, and in order to exploit their properties it is necessary to ensure that their magnetic state is retained on the on-surface 2D array. At this point, it is worth mentioning that recently great advances in the field of carbon-based π-magnetism have been reported thanks to the field of on-surface synthesis.^27^ In the past decade, the synthesis of various nanographenes (NGs) with open-shell ground state has been demonstrated, such as NGs with mono- and diradicals character and other high-spin states.^28−35^ Therefore, we envision that the synergistic combination of on-surface synthesis of radical building blocks and their noncovalent self-assembly is a promising strategy for designing large-scale 2D supramolecular organic radical frameworks.

In this work, we report the on-surface synthesis of a 2D hydrogen-bonded organic radical framework (2D-HBORF) on a Au(111) surface, achieved from a rationally designed tripodal starting material, **1**, that incorporates 7-azaindole building blocks to induce an extended 2D preorganization through hydrogen bond-directed self-assembly.^11^ This approach has been demonstrated to improve the stability of organic semiconductors in different devices.^36−38^ We report the on-surface synthesis of an unprecedented fluoradene-based compound **3**, with an intrinsic monoradical state, which assembles forming a 2D magnetic array. The rationalization of the reaction mechanism reveals that the formation of the radical compound **3** is driven by a single gold atom on the surface (adatom),^39−41^ providing suitable experimental conditions to yield the strained fluoradene system.^42,43^ Here, we demonstrate the controlled and scalable formation of large defect-free 2D-HBORFs on Au(111), ordered in a Kagome-honeycomb phase,^44,45^ in the size of hundreds of nanometers. We present an atomic scale characterization of the structural, electronic, and magnetic properties of this phase, which forms a unique HBORF, by means of scanning tunneling microscopy/spectroscopy (STM/STS) and noncontact atomic force microscopy (nc-AFM), complemented by density functional theory (DFT), and quantum mechanics/molecular mechanics (QM/MM) calculations together with molecular dynamics simulations (MDs).

## Results and Discussion

**Figure 1:**
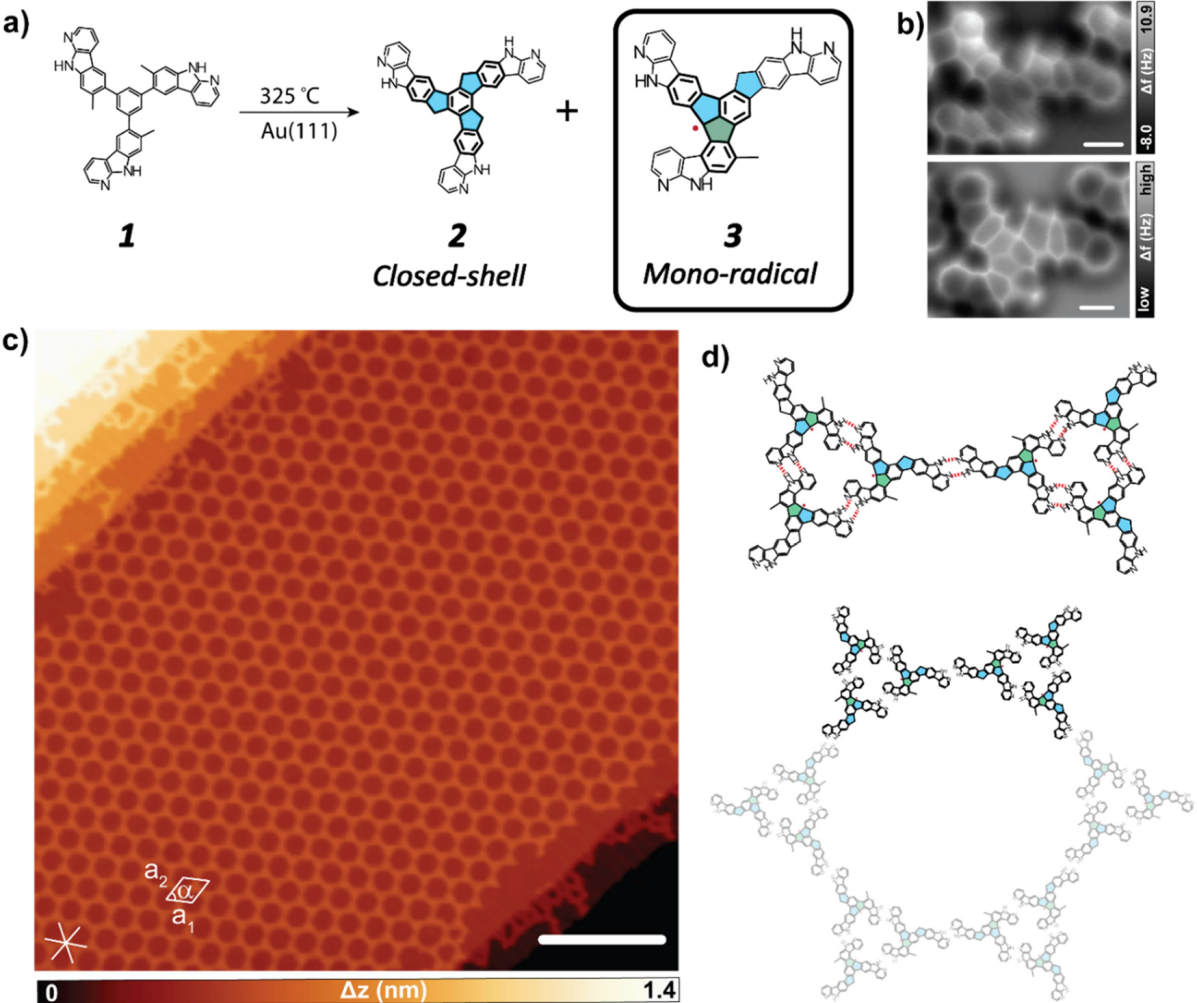
Structural characterization of **3**. (a) Synthetic route toward the formation of **2** and **3** on Au(111). (b) High-resolution nc-AFM image of **3** (*V*_b_ = 1 mV, scale bar = 4 Å) and simulated nc-AFM image of **3** on Au(111) (scale bar = 4 Å). (c) Constant-current overview STM image of the 2D-HBORF Kagome-honeycomb phase formed by product **3** (*V*_b_ = 100 mV, *I*_t_ = 20 pA, scale bar = 31 nm). The white crossed lines refer to the high symmetry directions of the Au(111). The unit cell of the lattice is defined by *a*_1_, *a*_2_, and α (*a*_1_ = *a*_2_ = 6.40 nm and α = 60°). (d) Molecular representation of the 2D-HBORF formed by **3**. The hydrogen bonds are represented as red dashed lines.

The synthesis of precursor 1,3,5-tris(7-methyl-α-carbolin-6-yl)benzene (**1**, see Figure 1a) was carried out according to the route depicted in Scheme S1. This molecule has been designed to have adequately located methyl groups that promote the subsequent on-surface cyclodehydrogenation and suitably oriented hydrogen bond donor and acceptor sites to direct the supramolecular self-assembly. The α-carboline precursor, **I**, was synthesized via nucleophilic aromatic substitution between *m*-toluidine and 2,3-dichloropyridine. Then, a palladium-catalyzed intramolecular coupling led to 7-methyl-α-carboline, **II**, which was subsequently brominated at position 6 to produce **III**. The NH group in compound **III** was Boc-protected, forming compound **IV**, prior to the triple Suzuki-Miyaura cross-coupling reaction with 1,3,5-tris(4,4,5,5-tetramethyl-1,3,2-dioxaborolan-2-yl)benzene to finally obtain the tripodal product **1** after the corresponding deprotection. All the intermediate and final products have been fully characterized by the usual spectroscopic techniques (See Supporting Information for experimental details).

Compound **1** was sublimed at 330 °C on the Au(111) surface kept at room temperature under ultrahigh-vacuum (UHV) conditions. Different assemblies stabilized by N–H···N bonds^11^ were observed due to the different conformations that **1** can adopt on the surface as a result of the rotation around the three σ bonds connecting the central benzene ring and the α-carboline units (Figure S1). The annealing of the intact precursor on Au(111) at 250 °C led to homochiral supramolecular assemblies ordered in a honeycomb structure with nanopores of 1.95 nm in diameter (see Figure S1b,c). The subsequent thermal annealing of the sample at 325 °C induces the oxidative ring-closure and cyclodehydrogenation reactions that promote the formation of the truxene core in product **2**, as well as the fluoradene core in product **3** (polyaromatic hydrocarbon cores are highlighted with thicker bonds in Figure 1a). A statistical analysis of the two reaction products indicates that, after annealing at 325 °C, 40% of the formed molecules correspond to product **2** and 60% to product **3** (statistics over ≈50,000 molecules). The truxene-based product, **2**, preserves the *C*_3h_ symmetry of the synthetic precursor **1**. Additionally, an alternative pathway leads to an asymmetric product, **3**, that contains a radical fluoradene core, as described in detail later.

Interestingly, the dissimilar orientation of the 7-azaindole units in products **2** and **3** results in different homochiral assemblies (see the comparison of both assemblies in Scheme S2). In both cases, each molecule sets six reciprocal hydrogen bonds through the three azaindole groups, with the adjacent molecules forming 2D supramolecular frameworks. Figure S2 shows an overview STM image where both assemblies coexist and reveals that the geometry of the supramolecular assemblies is significantly different. On the one hand, product **2** forms a hydrogen-bonded cyclic hexamer that expands, producing a honeycomb phase similar to the one formed by **1** (Scheme S2), with a nanopore size of 1.95 nm in diameter. The geometry of **2** is planar, and the presence of two hydrogens in the methylene group of the truxene core is observed by nc-AFM as bright protrusions, hampering any magnetic properties (Figure S3).^29,46^ On the other hand, the structure of **3** was also elucidated by nc-AFM measurements, as shown in Figure 1b. Product **3** consists of an extended fluoradene-based core fused to the 7-azaindole groups. This assignment is confirmed by the excellent matching between the experimental and simulated nc-AFM images shown in Figure 1b. Product **3** forms hydrogen-bonded cyclic trimers, which, in turn, self-assemble into macrocyclic dodecamer structures, producing a Kagome-honeycomb phase with nanopores of 5.00 nm in diameter and a unit cell defined by *a*_1_ = *a*_2_ = 6.40 nm and α = 60°, as shown in the overview STM image in Figure 1c and the molecular representation in Figure 1d.^47^ The Kagome-honeycomb phase is favored by the different directionality of the hydrogen bonds formed by the 7-azaindole groups in the asymmetric product **3**. It is worth mentioning that we find the Kagome-honeycomb phase forming defect-free large domains of hundreds of nanometers on the Au(111), as illustrated in Figure 1c, demonstrating the high quality and scalability of this supramolecular system.

To get more insight into the reaction pathway leading to the formation of products **2** and **3**, we carried out MD simulations with QM/MM partition to determine the free energy barriers of the dehydrogenation processes that trigger the on-surface synthesis catalyzed by a single gold adatom. The presence of adatoms is justified by previous surface diffusion studies on metal surfaces.^48,49^ In the case of Au(111) surface, the formation energy of Au adatoms was determined to be 0.79 eV,^50^ indicating an abundant amount of adatoms at the reaction temperatures.

**Figure 2:**
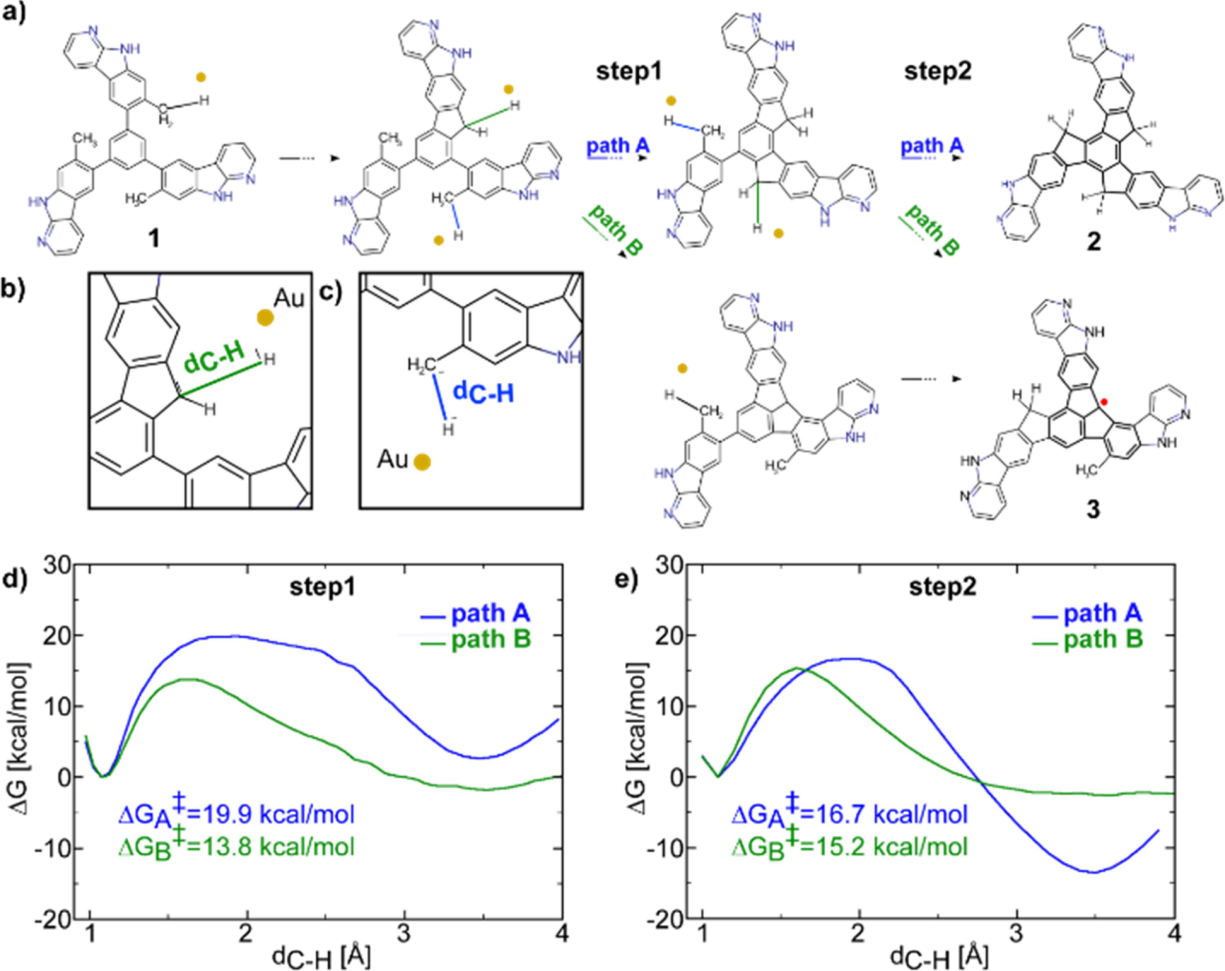
Theoretical analysis of two competing reaction pathways. (a) Simplified reaction scheme with relevant intermediates and products. Dashed arrows indicate the omission of reaction steps. For a full scheme, see Scheme S3. (b and c) Definition of the reaction coordinates for umbrella sampling simulations of fluorene and methyl dehydrogenations, respectively. (d and e) Free energy profiles of dehydrogenation of methyl (path A, blue) and fluorene (path B, green) in steps 1 and 2, respectively, catalyzed by a single gold adatom.

We propose a reaction mechanism (Scheme S3 and Figure 2), where the formation of **2** is determined by the cyclodehydrogenation involving the three methyl groups. In contrast, the formation of **3** is determined by the cyclodehydrogenation involving both methyl and fluorene groups at different stages of the reaction course mediated by a single Au adatom on the surface. Moreover, the proposed reaction pathway admits two bifurcations that lead to products **2** and **3**. Our calculations show that the different paths have similar low activation energies ∼14–20 kcal/mol (Figure 2b–e), rendering both pathways feasible, as is demonstrated by the experimental findings. Importantly, the gold adatom substantially lowers the activation energy required for the cleavage of the C–H bond at a given range of temperatures in the experiment conditions. It also partially passivates the radical character of the carbon atom, as illustrated in Figure S4, which substantially lowers the energy of the intermediate states. This underlines the fundamental role of the single gold adatom catalysis in the chemical transformation.

The synthetic route necessarily starts with dehydrogenating one of the methyl groups, followed by the reaction with the central benzene ring to produce the cyclization of the first fluorene-like substructure, see scheme in Figure 2a. This process can occur twice more to obtain compound **2** (path A). Regarding compound **3**, after the first cyclodehydrogenation, the free energy profiles corresponding to the bifurcation identified as step 1 (Figure 2a,d) exhibit a higher probability for the reaction following a reaction pathway B, through the dehydrogenation of fluorene (Figure 2b). The free activation energy of this step is 6 kcal/mol lower than the dehydrogenation of the methyl group (path A and Figure 2c). In the second step (Figure 2e), the barriers at the bifurcation are nearly identical, with a difference of 1.5 kcal/mol, again in favor of path B. To gain a better understanding of the postulated mechanism, we further investigated the free energy profiles of the possible consecutive reaction steps immediately after the first bifurcation, namely the cyclization or dissociation steps (Figure S5a–c) catalyzed by a single Au adatom on the surface. Based on the obtained results, Figure S6 summarizes the proposed reaction sequence with the corresponding free energy profiles and consists of the following paths.

On the one hand, after dehydrogenating the fluorene (path B, green), a second dehydrogenation process leads to a carbenoid intermediate stabilized by a single Au adatom. In the subsequent step, the fluoradene unit is formed, and finally, the last cyclodehydrogenation process gives rise to the formation of **3**. On the other hand, the dehydrogenation of the methyl group (path A, blue) forms an indenofluorene core (intermediate species IM3 blue in Figure S6b). Afterward, a double dehydrogenation process leads to a carbenoid intermediate passivated by the Au adatom that results in the formation of **3**. Overall, the similar dehydrogenation energy barriers in specific steps explain the bifurcation of the reaction and the coexistence of both phases, supporting the experimental findings. Here, we argue that the higher barrier in the second dissociation step on path B forming carbenoid species is still accessible at the reaction conditions. Notably, the radical character of the carbenoid group is passivated by surface Au adatoms, which energetically stabilizes the intermediate IM3 with respect to a possible cyclization product (compare Figure S5a,b). Consequently, the formation of **3** is decided solely by the first dissociation at the bifurcations.

**Figure 3:**
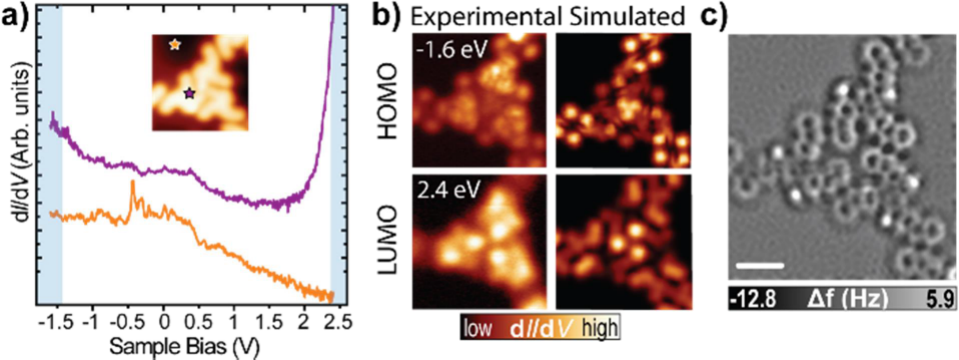
Electronic characterization of **3**. (a) Differential conductance d*I*/d*V* spectra acquired over **3** and on the Au(111) as reference. The spectra positions are depicted in the inset STM image. (b) Experimental and simulated d*I*/d*V* maps showing the HOMO and LUMO of a trimer structure formed by **3**. (c) Nc-AFM image of a trimer on Au(111), which corresponds to the same trimer illustrated in (b) (scale bar = 8.5 Å).

We performed STS measurements to understand the electronic properties of **3** on Au(111). The differential conductance d*I*/d*V* spectrum acquired on top of **3** reveals two orbital resonances at −1.4 and 2.4 eV, as shown in Figure 3a. The d*I*/d*V* maps acquired at the specific energies of the two orbital resonances show a characteristic spatial distribution that nicely matches with the simulated d*I*/d*V* maps of the highest occupied molecular orbital (HOMO) and lowest unoccupied molecular orbital (LUMO), respectively, as illustrated in Figure 3b. Thus, we can conclude that the resonances at −1.4 and 2.4 eV correspond to the HOMO and LUMO orbitals of **3**, respectively, and that **3** has an experimental energy gap of 3.8 eV, in agreement with the calculated HOMO–LUMO gap of 3.9 eV (Figure S7).

**Figure 4:**
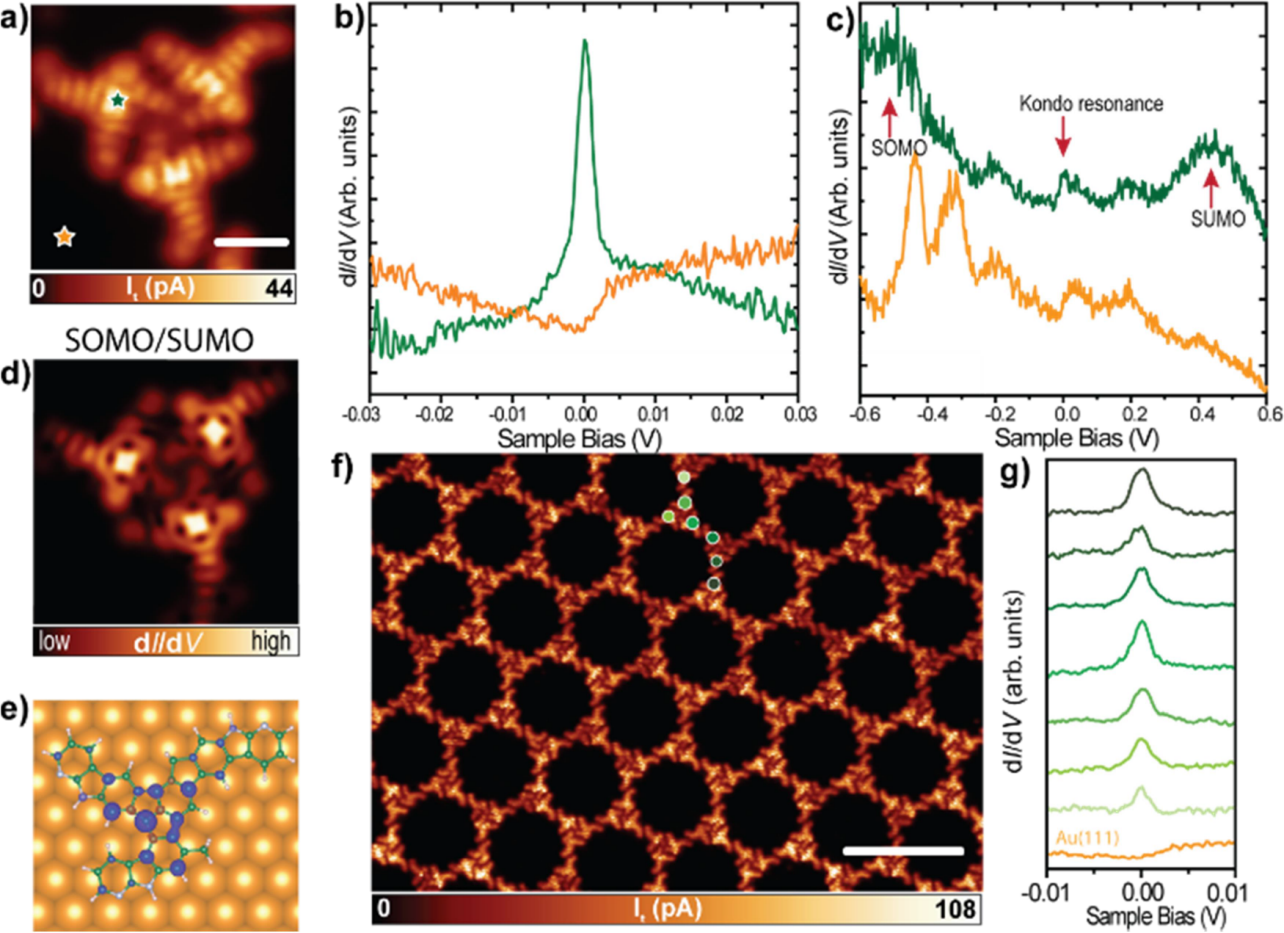
Characterization of the magnetic properties of **3** on Au(111). (a) Constant-height STM image of **3** at low bias voltage revealing the enhancement in the LDOS around the Fermi energy (*V*_b_ = 1 mV, scale bar = 1 nm). (b) Low-energy and (c) medium-energy d*I*/d*V* spectra acquired on **3**. The positions of the spectra are depicted in (a) by colored stars. (d) Simulated d*I*/d*V* map of the SOMO/SUMO (e) DFT calculated spin density. (f) Overview constant-height STM image of the Kagome-honeycomb phase, confirming the existence of magnetic states in all the molecular building blocks (*V*_b_ = 5 mV, scale bar = 8 nm). (g) Short-range d*I*/d*V* spectra acquired over a series of 7 adjacent molecules confirming the existence of a Kondo resonance in all molecules.

Then, to rationalize the magnetic properties of **3**, we performed STM/STS measurements at low bias voltages. Figure 4a shows a constant-height STM image of **3** acquired at 1 meV with a CO-functionalized tip. Interestingly, product **3** features characteristic bright lobes at this bias voltage due to an enhancement in the local density of states close to the Fermi energy. Then, we recorded STS in a lower energy bias window, revealing the emergence of a strong peak centered at the Fermi energy, which is assigned to a Kondo resonance (see Figure 4b) characteristic from the screening effect of a magnetic moment with the conduction electrons of the Au(111).^51,52^ Furthermore, we could identify two additional resonances at −0.5 and 0.5 eV, as shown in Figure 4c. Tentatively, we assigned such resonances to the singly occupied molecular orbital (SOMO) and singly unoccupied molecular orbital (SUMO), respectively. We performed spin-unrestricted DFT calculations to corroborate the experimental STS measurements, predicting one unpaired electron hosted in a SOMO orbital (see Figure S7). The simulated SOMO/SUMO map shown in Figure 4d resembles very well the constant-height STM image in Figure 4a. Figure 4e illustrates the calculated unpaired spin density of **3** on Au(111). Altogether, we can conclude that **3** possesses an intrinsic paramagnetic moment *S* = 1/2, which is screened by the free electrons of the metal to give rise to the Kondo effect. The absence of spin-excitation signal in d*I*/d*V* spectra discards the existence of spin–spin interactions between adjacent molecules.

According to the experimental observations, product **3** spontaneously adopts an open-shell form, and it does not react with any residual gas present in the UHV chambers,^53^ suggesting its inertness. To theoretically rationalize the presence of the radical character of product **3** versus its closed-shell counterpart, we compared the thermodynamic stability of the open-shell form with respect to the closed-shell alternative, which consists of a hydrogen-passivated fluoradene (see Figure S8). According to total energy DFT calculations, both the hydrogen transfer from the fluoradene unit to a gold cluster and the formation of molecular hydrogen in the presence of a free hydrogen atom in the UHV chamber,^54^ are thermodynamically more favorable, see Figure S8 and Table S1, explaining the radical character of **3**.

Finally, we investigated the magnetic properties in a larger area to corroborate the long-range magnetic order. To this aim, we took different overview constant-height STM images of the Kagome-honeycomb phase at a low bias voltage. Figure 4f shows a representative STM image demonstrating that the magnetic character of **3** is a phenomenon extended throughout the supramolecular assembly. In Figure 4g we can observe the characteristic Kondo resonance in the d*I*/d*V* spectra acquired over a series of 7 adjacent molecules from Figure 4f. This denotes the monoradical character of all the molecules integrated on the hundreds of nanometers extended assembly. Altogether, we demonstrate the synthesis of a 2D-HBORF formed by product **3** on a large scale.

## Conclusions

In summary, we have presented the growth of the radical hydrogen-bonded supramolecular framework by combining the rational design and synthesis of a suitable tripodal precursor and its subsequent chemical transformation assisted by a single gold adatom on the surface. The novel π-expanded polyheteroaromatic system with a fluoradene core has provided an unprecedentedly stable organic radical with an inherent spin state 1/2 on the Au(111) substrate. The integration of 7-azaindole units in the molecular building blocks with an open-shell topology forms large homochiral assemblies stabilized by N–H···N bonds that constitute a unique 2D-HBORF. Our work represents an interdisciplinary study combining supramolecular chemistry, π-magnetism, and on-surface synthesis, contributing to the development of supramolecular organic radical chemistry. We envision that this work may stimulate the synthesis of novel supramolecular organic radical frameworks with important applications in, among others, sensors, encapsulation, and quantum technology or catalytic redox reactions.

## Supporting Information Available

Experimental details, synthesis, characterizations, computational studies, and NMR spectra (PDF)
