# Supplementary material for: On-Surface Synthesis of a Radical 2D Supramolecular Organic Framework
Source: J Am Chem Soc. 2024 Jan 25;146(5):3531–8. doi: 10.1021/jacs.3c13702 (PMC10859929; doi:10.1021/jacs.3c13702)
Supplement: Supplementary file 1 — ja3c13702_si_001.pdf [file ja3c13702_si_001.pdf]

Supplementary information

On-surface synthesis of a radical 2D supramolecular organic framework

Federico Frezza,[†,‡,♠] Adam Matěj,[†,#,♠] Ana Sánchez-Grande,[†,*] Manuel Carrera,[ξ] Pingo Mutombo,[†,η] Manish Kumar,[†] David Curiel,[ξ,*] Pavel Jelínek[†, ψ,*]

[†] Institute of Physics of Czech Academy of Sciences. Cukrovarnická 10, 16200 Prague 6, Czech Republic.

[‡] Faculty of Nuclear Sciences and Physical Engineering, Czech Technical University in Prague Břehová 78/7, 11519 Prague 1, Czech Republic.

[#] Department of Physical Chemistry, Faculty of Science, Palacký University, 17. Listopadu 12, 779 00, Olomouc, Czech Republic.

[ξ] Department of Organic Chemistry, University of Murcia Campus of Espinardo, 30100 Murcia, Spain.

[η] Département de Raffinage et Pétrochimie, Faculté de Pétrole, Gaz et Énergies Renouvelables, Université de Kinshasa, BP 127, Kinshasa XI, République Démocratique du Congo.

[ψ] CATRIN-RCPTM, Palacký University, Šlechtitelů 27, 783 71, Olomouc, Czech Republic.

♠ These authors contributed equally.

* Authors to whom correspondence should be addressed

Methods

STM and nc-AFM experiments

Experiments were performed in custom-designed ultra-high vacuum systems (base pressure below 5 x 10^{-10} mbar) hosting a commercial low-temperature microscope with STM/AFM capabilities Createc GmbH. The Au(111) substrate (MaTeck GmbH) was cleaned by repeated cycles of Ar^+ ion sputtering (E = 1 keV) and subsequent annealing to 740 K for 10 minutes. Unless otherwise noted, All STM images were taken in constant-current mode at a sample temperature of 5.0 K. Scanning parameters are specified in each figure caption. Molecular precursor **1** was thermally sublimed onto the clean Au(111) surface kept at RT with (sublimation temperatures of 330°C). Non-contact AFM measurements were performed with Pt/Ir tip attached to a Qplus tuning fork[1] sensor from Createc. The tip was *a posteriori* functionalized by controlled adsorption of a single CO molecule at the tip apex from a previously CO-dosed surface.[2] The sensors were driven at their resonance frequency (30 kHz) with a constant amplitude of ~ 60/50 pm. The frequency shift from the resonance of the sensor (with the attached CO-functionalized tip) was recorded in a constant-height mode (Nanonis SPM for Createc GmbH). The STM and nc-AFM images were analyzed using WSxM).[3]

Computational details

Free energy molecular dynamic simulations with umbrella sampling were carried out using QM/MM partition. We used Sander from Ambertools, which is a part of the Amber20 suite as the MD engine and overhead for the simulations.[4] For the QM part, we employed FIREBALL as a fast DFT code for the relaxation and electronic structure of the QM partition.[5] The QM part includes the molecule and a gold adatom, while the Au(111) surface is modeled at the MM level, interacting only via vdW forces. The vdW parameters for the gold atoms were obtained from the Interface force field.[6] The protocol consists of relaxation of the initial state, elongation of the bond along the defined reaction coordinate, and minimization in each step. Each structure was then tempered in the relevant window to the final temperature, and a production run at constant temperature was carried out subsequently. The umbrella sampling windows were spaced by 0.1 Å, with a time step of 0.5 fs and simulation time of 10 ns, and the overlap of sampling in between windows was checked. The resulting sampled bond distances were analyzed using the weighted histogram analysis method, obtaining free energy profiles.[7,8]

Synthetic procedure

Reagents and solvents used for the synthesis were commercial suppliers and were used without further purification. Unless stated otherwise, the reactions were carried out under nitrogen atmosphere. Column chromatography was run with silica gel 60 A CC 70-200 µm as stationary phase. ^{1}H-NMR and ^{13}C-NMR spectra were recorded at room temperature with a Bruker AV400 or AV300 spectrometers. Chemical shifts are referred to the residual peak of the deuterated solvent. Mass spectra were measured with an HPLC-MS TOF 6220 instrument.

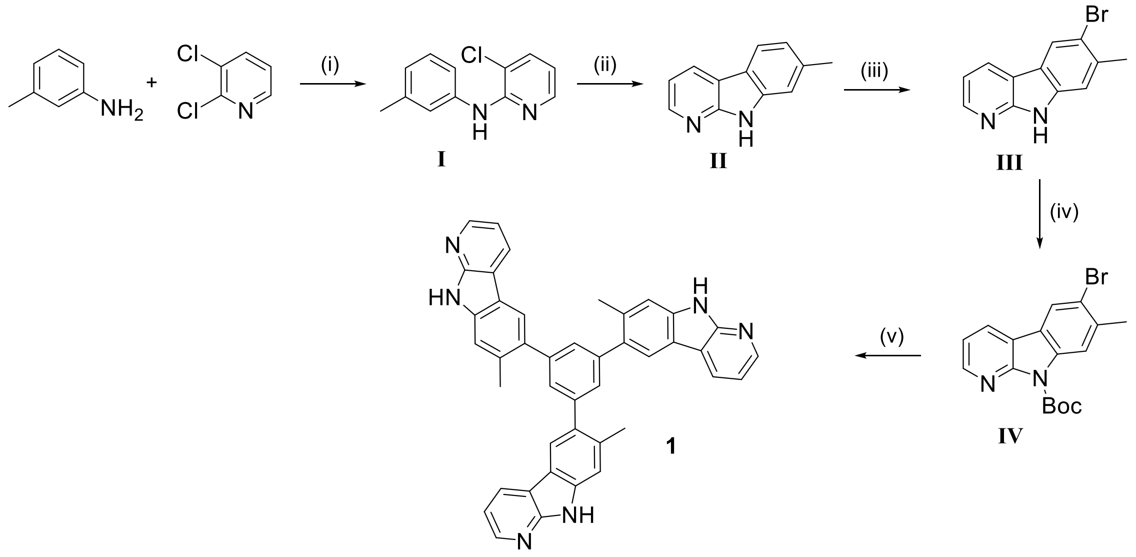

(i) 170ºC (98%); (ii) $Pd(dba)_2$, $PCy_3{\cdot}HBF_4$, DBU, dioxane, 180ºC (67%); (iii) NBS, CH_2Cl_2, 0 ºC (86%); (iv) Boc_2O, DMAP, acetonitrile, r.t. (91%); (v) 1) 1,3,5-tris(4,4,5,5-tetramethyl-1,3,2-dioxaborolan-2-yl)benzene, (dppf)$PdCl_2$, $CsCO_3$, dioxane, H_2O, 90ºC; 2) TFA, CH_2Cl_2, r.t. (51%)

Scheme S1. Synthetic route toward compound **1**.

3-chloro-2-(*m*-methylphenylamino)pyridine, I: A round bottom flask equipped with a reflux condenser was charged with 1,2-dichloropyridine (7.42 g, 50 mmol) and *m*-Toluidine (16 mL, 150 mmol). The mixture was heated at 170 °C for 2 hours. After cooling down, the crude was dissolved in CH_2Cl_2 (100 ml) and washed with Na_2CO_3 (aq., sat.) (3x50 ml). The organic phase was dried over Na_2SO_4, filtered and the solvent was evaporated under reduced pressure. The crude was purified by column chromatography, using hexane/ethyl acetate (9/1) as eluent, to yield 3-chloro-2-(m-methylphenylamino)pyridine (10.1 g, 98% yield) as a dark red oil.

^{1}H-NMR (400 MHz, $CDCl_3$), δ (ppm): 8.01 (dd, J = 4.9, 1.6 Hz, 1H), 7.41 (dd, J = 7.7, 1.6 Hz, 1H), 7.34 (d, J = 8.0 Hz, 1H), 7.31 (s, 1H), 7.11 (t, J = 7.8 Hz, 1H), 6.81 (s, 1H), 6.73-6.78 (m, 1H), 6,54 (dd, J = 7.7, 4.9, 1H), 2.25 (s, 3H). ^{13}C-NMR (101 MHz, $CDCl_3$), δ (ppm): 151.4, 145.8, 139.6, 138.7, 136.6, 128.8, 123.7, 120.6, 117.2, 116.1, 115.3, 21.6. HRMS (ESI), *m/z*, $[M+H]^+$, calc. for $C_{12}H_{12}ClN_2$: 219.0684, found: 219.0694.

7-methyl-α-carboline, II: A mixture of 3-chloro-2-(*m*-methylphenylamino)pyridine (4.93 g, 23 mmol), $Pd(dba)_2$ (4 mol%), $PCy_3·HBF_4$ (8 mol%) and DBU (8.8 mL, 58 mmol) in dry 1,4-dioxane (100 mL) was bubbled with nitrogen for 20 minutes. Then, this mixture was placed in a sealed tube and heated in an oven at 180 °C for 3 days. Once the reaction cooled down, the solvent was evaporated under reduced pressure. The dark crude was purified by column chromatography using dichloromethane/ethyl acetate (3/1) as eluent. The product was obtained as an analytically pure yellow solid (2.8 g, 67% yield).

^{1}H-NMR (300 MHz, $CDCl_3$), *δ* (ppm): 10.93 (s, 1H), 8.49 (dd, *J* = 5.0, 1.6 Hz, 1H), 8.31 (dd, *J* = 7.7, 1.6 Hz, 1H), 7.94 (d, *J* = 8.0 Hz, 1H), 7.35-7.37 (m, 1H), 7.20 (dd, *J* = 7.7, 5.0 Hz, 1H), 7.09-7.13 (m, 1H), 2.56 (s, 3H). ^{1}H-NMR (300 MHz, DMSO), *δ* (ppm): 11.70 (s, 1H), 8.35-8-42 (m, 2H), 8.00 (d, *J* = 8.0 Hz, 1H), 7.28-7.34 (m, 1H), 7.15 (dd, *J* = 7.6, 4.9 Hz, 1H), 7.00-7.06 (m, 1H), 2.46 (s, 3H). ^{13}C-NMR (75 MHz, DMSO-d_6), *δ* (ppm): 152.0, 145.4, 139.3, 136.3, 127.8, 120.9, 120.9, 118.1, 115.5, 114.8, 111.2, 21.8. HRMS (ESI), *m/z*, $[M+H]^+$, calc. for $C_{12}H_{11}N_2$: 183.0917, found: 183.0921.

6-bromo-7-methyl-α-carboline, III: *N*-Bromosuccinimide (0.37 g, 2 mmol) was added portionwise to a stirred suspension of 7-methyl-α-carboline (0.37 g, 2 mmol) in CH_2Cl_2 (20 mL) at 0 °C. Stirring was continued for 1 day. The solvent was evaporated under reduced pressure and the crude was triturated with methanol. A white solid was collected by filtration to obtain 0.46 g of 6-bromo-7-methyl-α-carboline (86 %).

^{1}H-NMR (300 MHz, DMSO-d_6), *δ* (ppm): 11.80 (s, 1H), 8.43 (d, *J* = 7.5 Hz, 1H), 8.30-8.39 (m, 2H), 7.41 (s, 1H), 7.13 (dd, *J* = 7.6, 4.9 Hz, 1H), 2.44 (s, 3H). ^{13}C-NMR (75 MHz, DMSO-d_6), *δ* (ppm): 152.2, 146.4, 138.3, 135.0, 128.7, 124.4, 120.4, 115.3, 114.7, 114.4, 113.1, 23.4. HRMS (ESI), *m/z*, $[M+H]^+$, calc. for $C_{12}H_{10}BrN_2$: 261.0022, found: 261.0026.

2-bromo-7-methyl-9-*tert*-butoxycarbonyl-α-carboline, IV: A round bottom flask was charged with 6-bromo-7-methyl-α-carboline (0.26 g, 1 mmol), di-*tert*-butyl dicarbonate (0.95 g, 4.4 mmol), 4-(dimethylamino)pyridine (0.09 g, 0.8 mmol) and acetonitrile (10 mL). The orange mixture was stirred at room temperature overnight. Then, the solvent was evaporated under reduced pressure and the crude was purified by column chromatography using hexane/ethyl acetate (4/1) as eluent. The product was isolated as a white solid (0.33 g, 91 %).

^{1}H-NMR (400 MHz, $CDCl_3$), *δ* (ppm): 8.60 (dd, *J* = 4.9, 1.7 Hz, 1H), 8.13 (s, 1H), 8.13 (dd, *J*= 7.7, 1.7 Hz, 1H), 8.06 (s, 1H), 7.27 (dd, *J* = 7.7, 4.9 Hz, 1H), 2.55 (s, 3H), 1.77 (s, 9H). ^{13}C-NMR (75 MHz, $CDCl_3$), *δ* (ppm): 151.6, 149.5, 147.5, 137.5, 136.9, 127.9, 123.7, 122.2, 119.3, 118.9, 118.2, 117.8, 84.8, 28.4, 24.4. HRMS (ESI), *m/z*, $[M+H]^+$, calc. for $C_{17}H_{18}BrN_2O_2$: 361.0546, found: 361.0554.

1,3,5-tris(7-methyl-α-carbolin-6-yl)benzene, 1: A Schlenk flask was loaded with compound IV (0.62 g, 1.7 mmol), 1,3,5-tris(4,4,5,5-tetramethyl-1,3,2-dioxaborolan-2-yl)benzene (0.18 g, 0.5 mmol), (dppf)$PdCl_2$ (10 mol%) and Cs_2CO_3 (1.65 g, 5 mmol). Inert atmosphere was made through vacuum/nitrogen cycles. Another mixture of water (3 mL) and 1,4-dioxane (15 mL) was bubbled with nitrogen for 20 minutes and added to the Schlenk flask. The reaction was stirred at 90 °C for three days. After cooling down, water was added

to the reaction flask and the resulting slurry was filtered to obtain a white solid that was purified by column chromatography using dichloromethane/tetrahydrofurane (95/5 to 1/3 gradient) as eluent. The isolated pale yellow solid was deprotected by stirring with trifluoroacetic acid (0.8 mL, 10 mmol) in CH_2Cl_2 (3 mL) at room temperature. The final product was isolated as an off-white solid (0.13 g, 51 %).

^{1}H-NMR (300 MHz, DMSO-d_6), δ (ppm): 11.75 (s, 1H), 8.50 (dd, J = 7.7, 1.3 Hz, 1H), 8.37 (dd, J = 4.8, 1.6 Hz, 1H), 8.20 (s, 1H), 7.46 (s, 2H), 7.16 (dd, J = 7.7, 4.9 Hz, 1H), 2.57 (s, 3H). ^{13}C-NMR (75 MHz, DMSO-d_6): 152.3, 145.7, 141.7, 138.5, 134.1, 133.5, 128.9, 128.3, 122.3, 118.8, 115.5, 114.9, 112.3, 21.5. HRMS (ESI), *m/z*, $[M+H]^+$, calc. for $C_{42}H_{31}N_6$: 619.2605, found: 619.2628.

^{1}H-NMR (400 MHz, $CDCl_3$) of compound **I**.

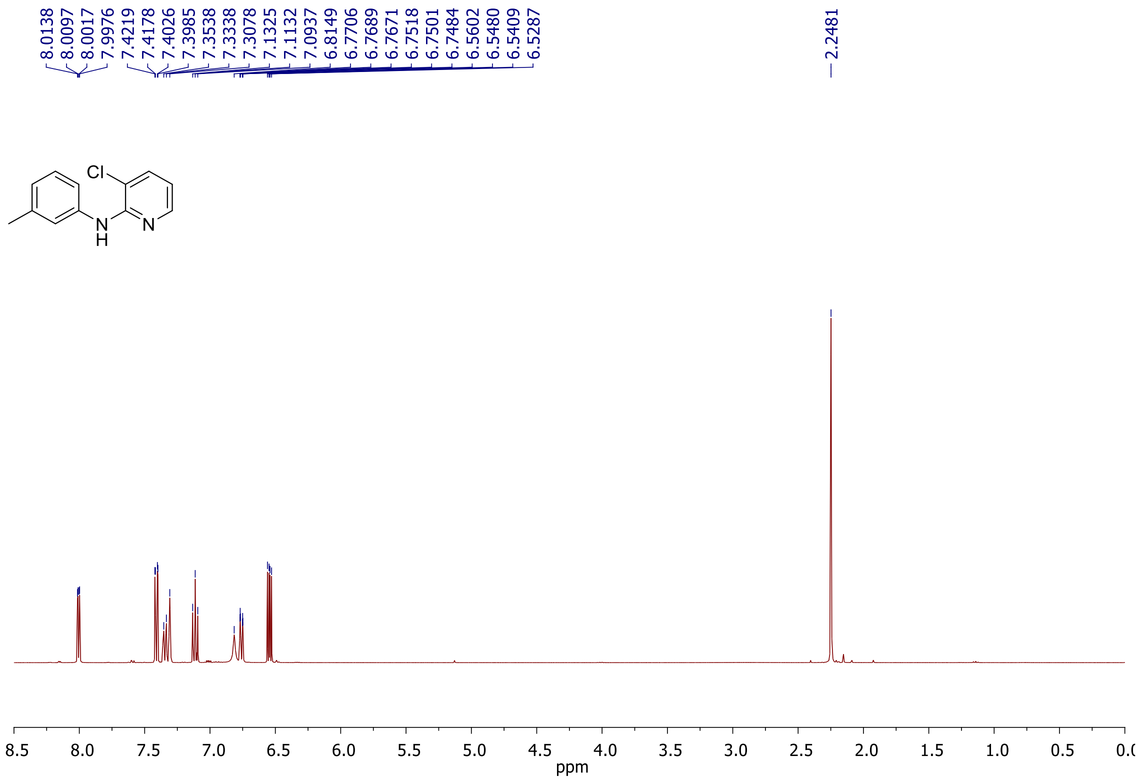

^{13}C-NMR (101 MHz, $CDCl_3$) of compound **I**.

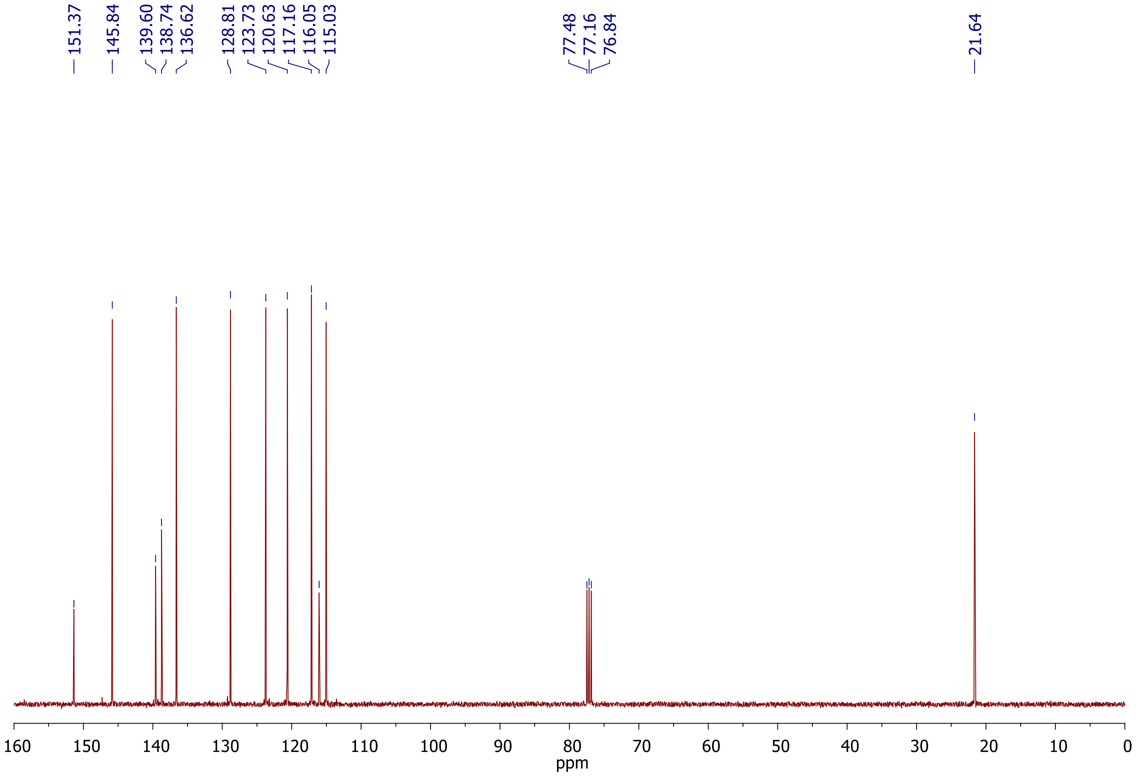

^{1}H-NMR (300 MHz, DMSO-d_6) of compound **II**.

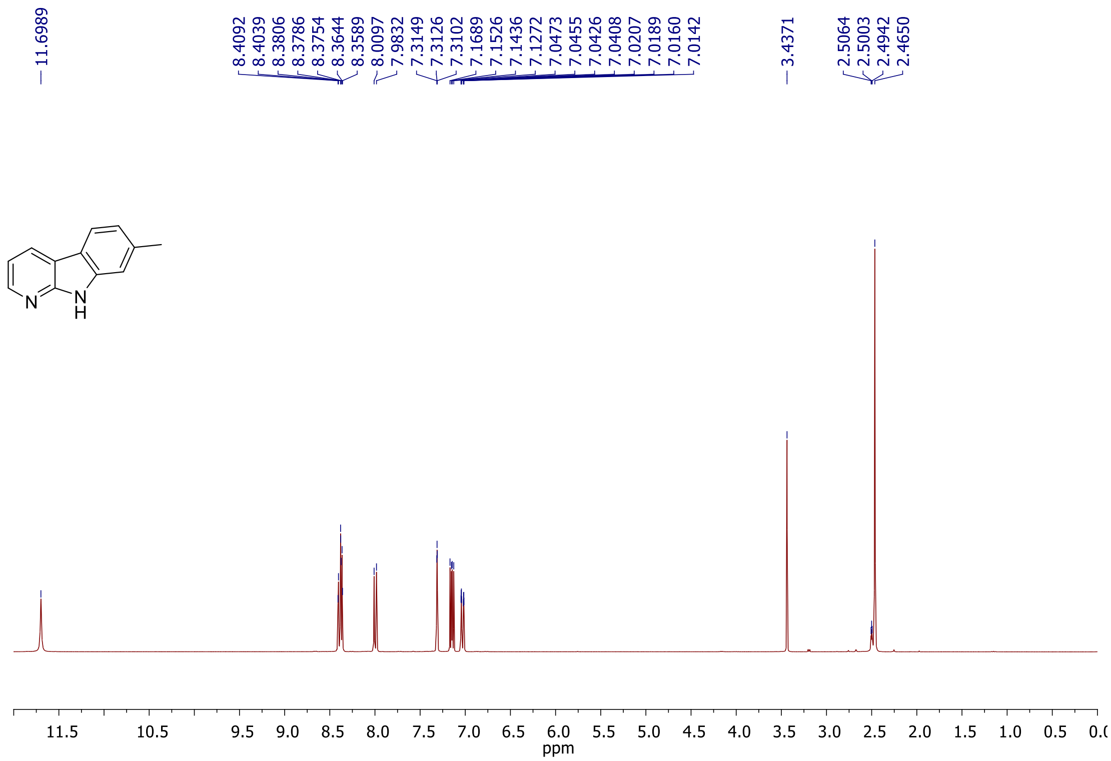

^{13}C-NMR (75 MHz, DMSO-d_6) of compound **II**.

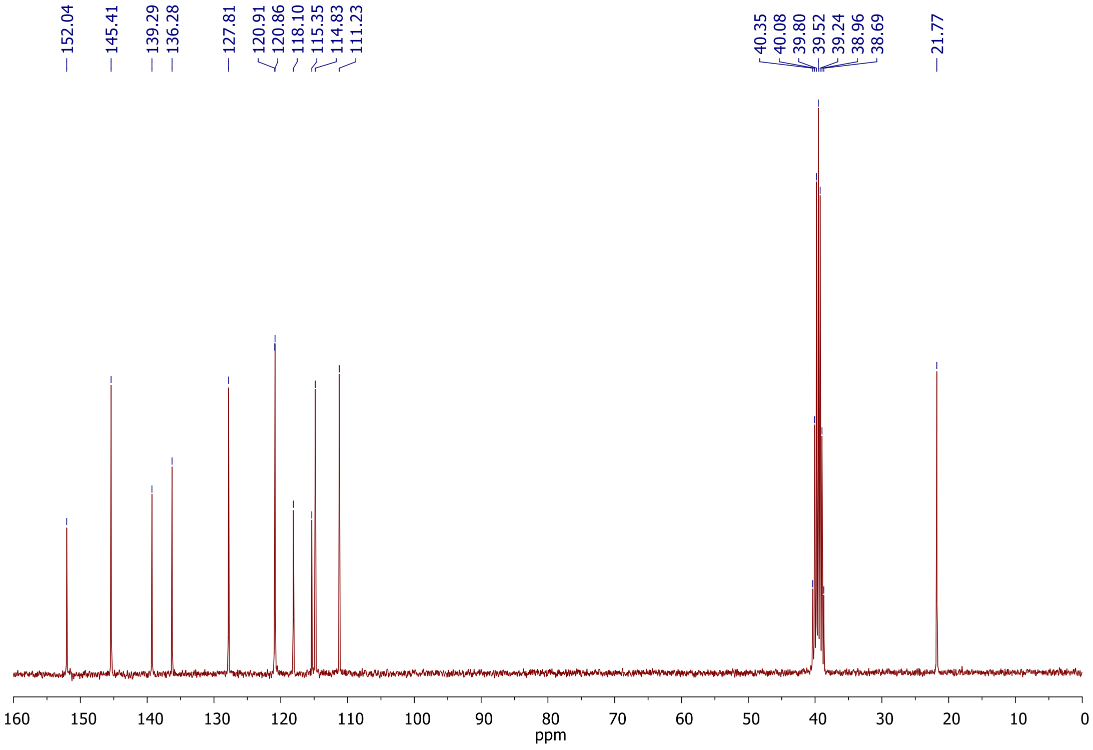

^{1}H-NMR (300 MHz, DMSO-d_6) of compound **III**.

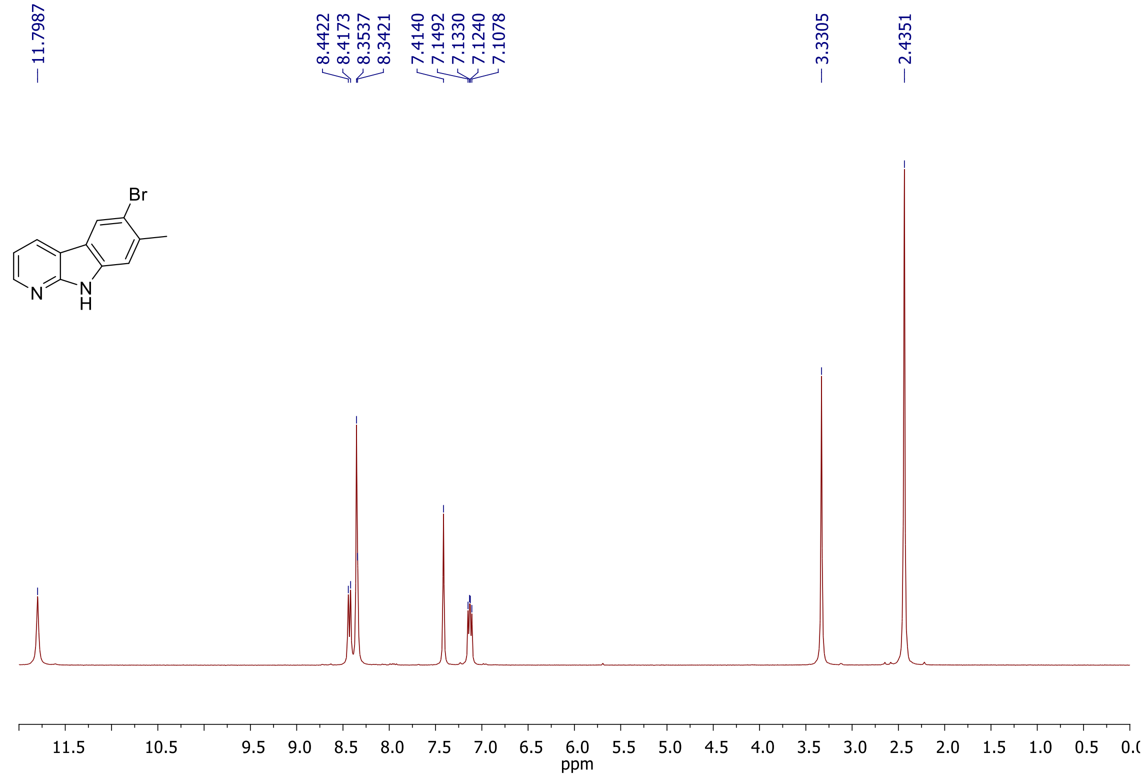

^{13}C-NMR (75 MHz, DMSO-d_6) of compound **III**.

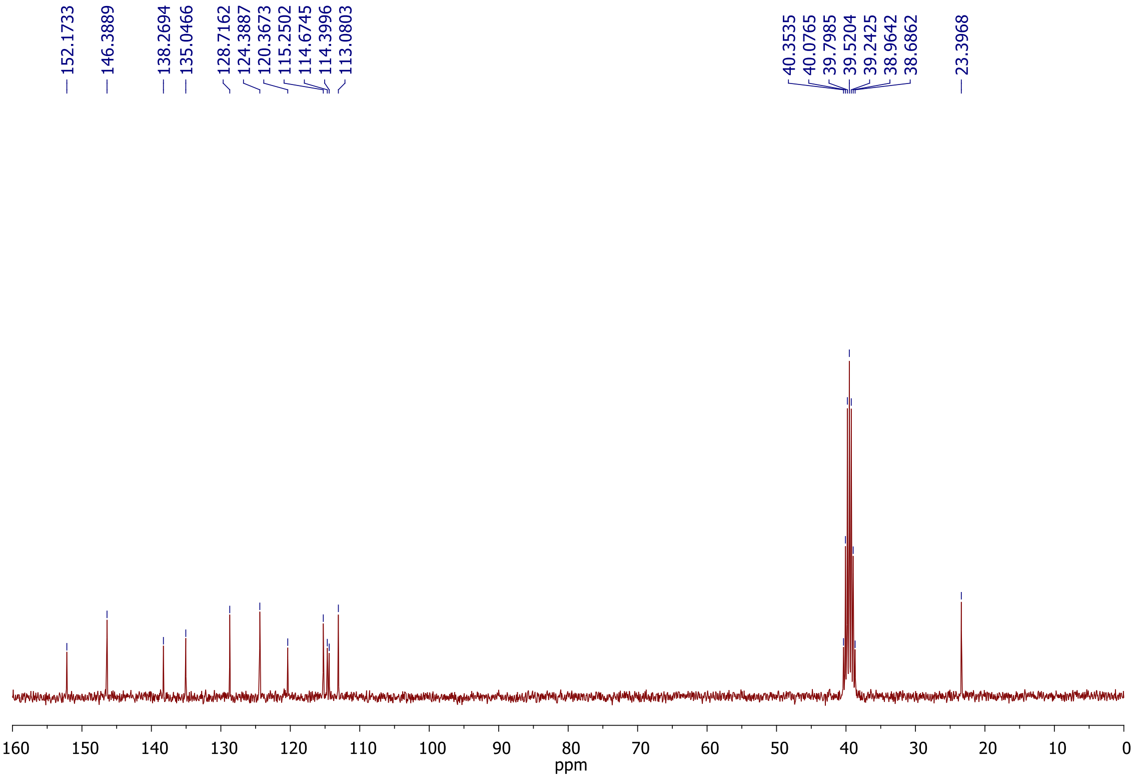

^{1}H-NMR (400 MHz, $CDCl_3$) of compound **IV**.

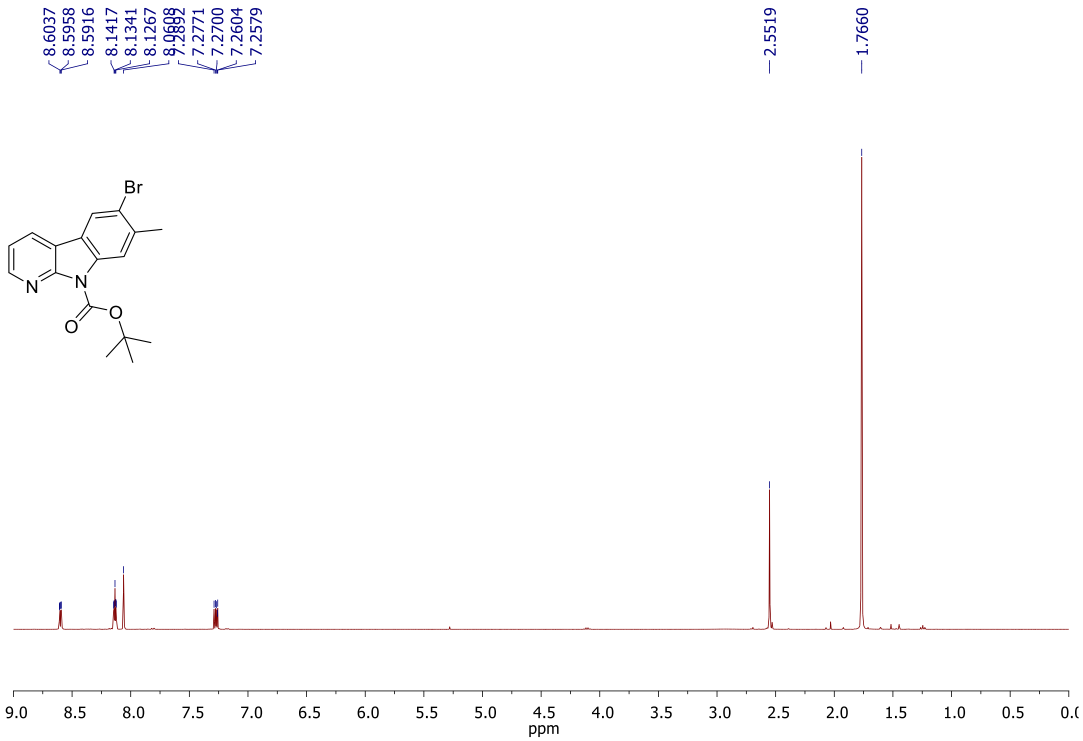

^{13}C-NMR (75 MHz, $CDCl_3$) of compound **IV**.

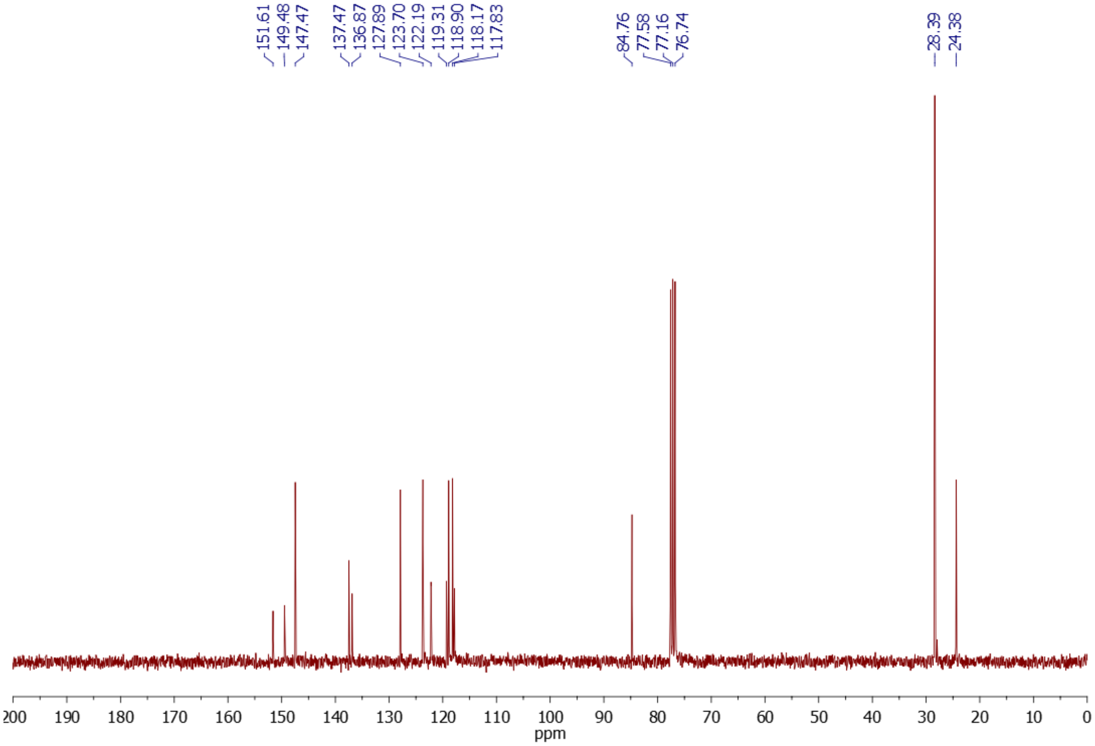

^{1}H-NMR (300 MHz, DMSO-d_6) of compound ***1***.

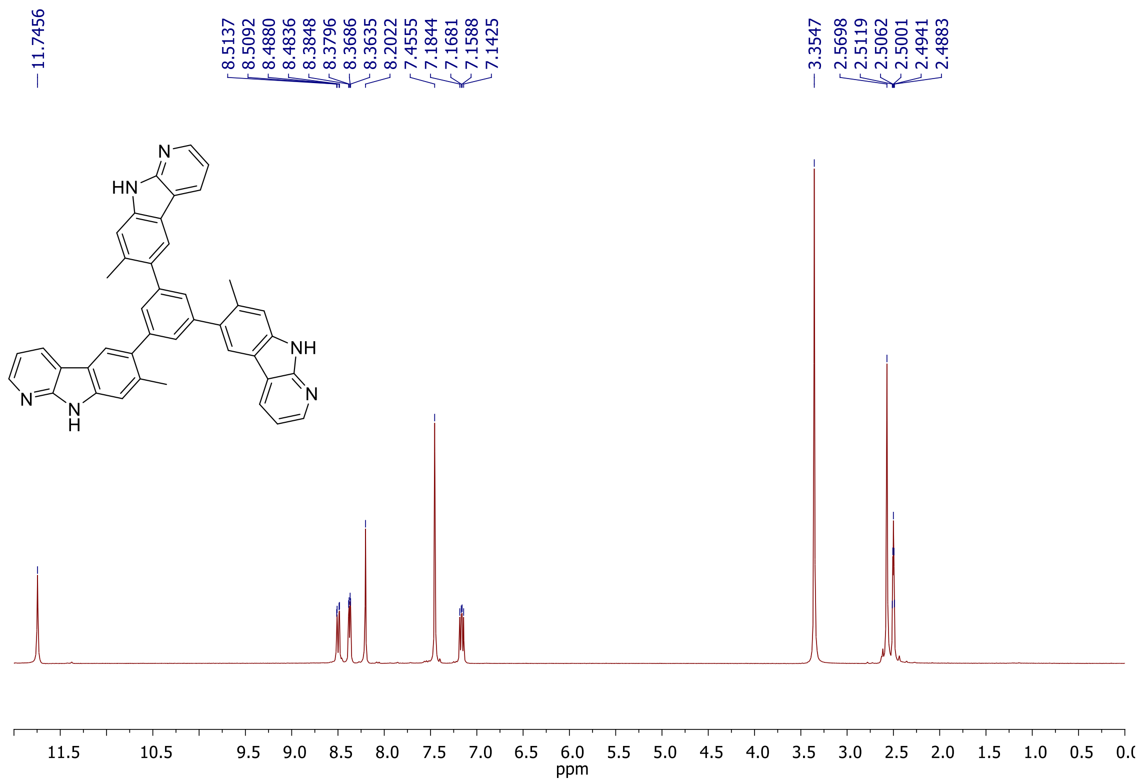

^{13}C-NMR (75 MHz, DMSO-d_6) of compound ***1***.

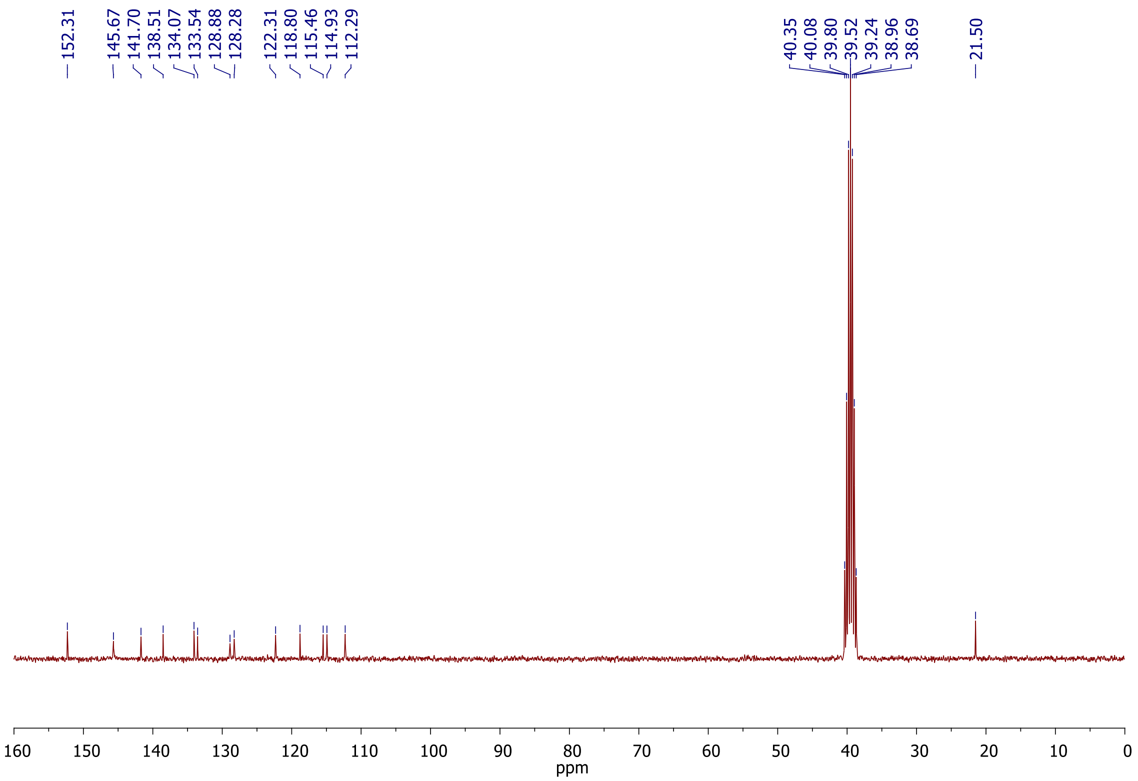

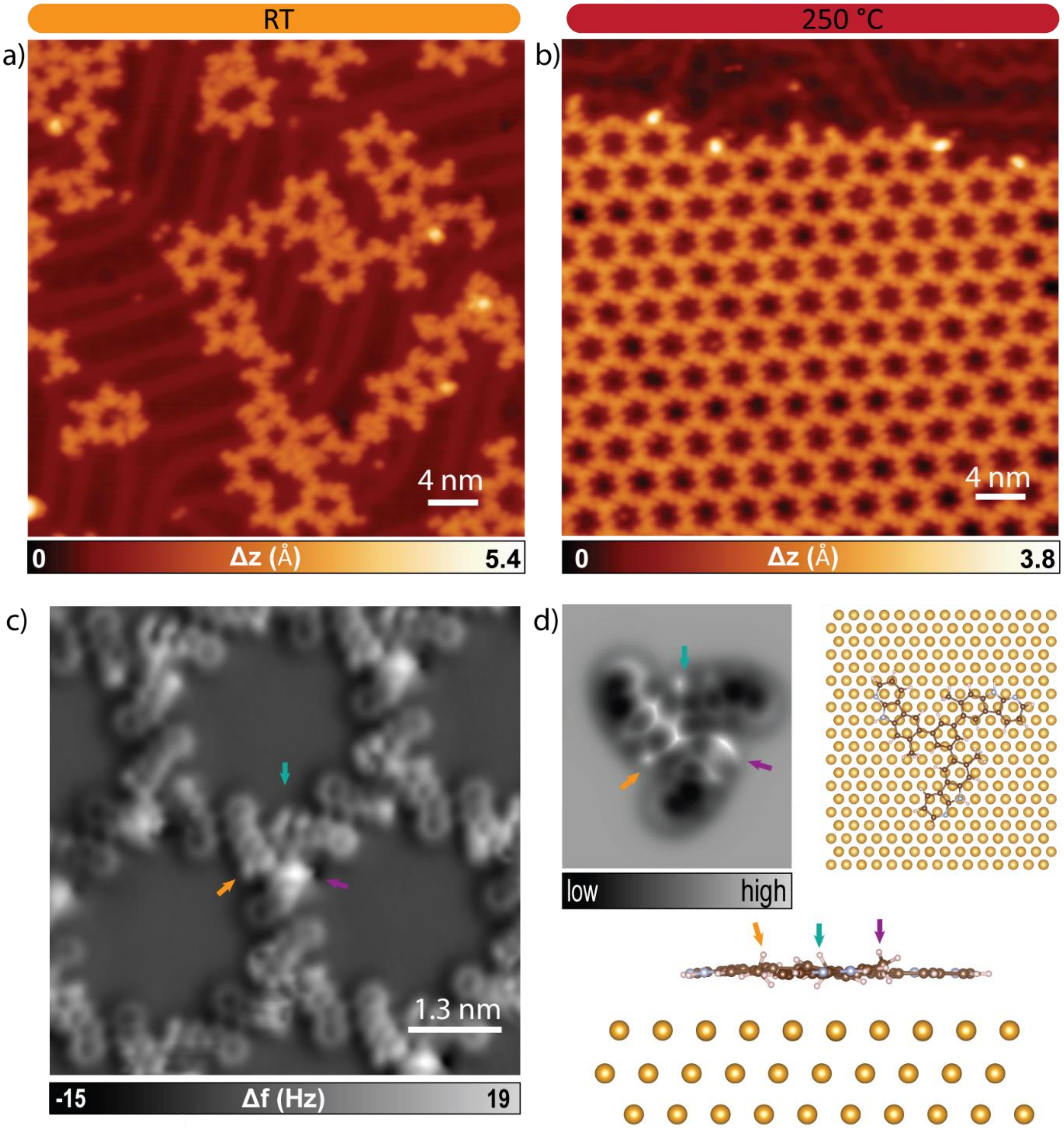

Figure S1. Deposition of **1** on the Au(111) substrate at RT and subsequent annealing at 250 °C. (a) Overview STM image of **1** adopting different configurations after deposition on the Au(111) crystal kept at RT (V_b = 200 mV, I_t = 100 pA) . (b) Overview STM image after annealing at 250 °C forming homochiral assemblies of **1** (V_b = 100 mV, I_t = 200 pA). (c) Nc-AFM image after the annealing at 250 °C revealing the non-planar geometry of **1** with one methyl group protruding (purple arrow), other in plane (orange arrow) and the third one slightly pushed down (cyan arrow) (V_b = 1 mV). (d) DFT equilibrium geometry of **1** on Au(111) confirming the non-planar geometry.

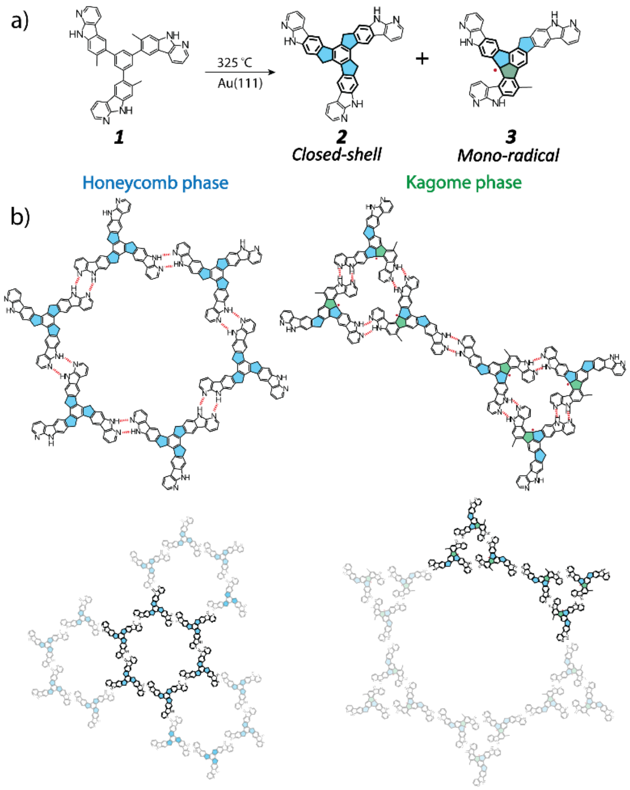

Scheme S2. a) Synthesis of closed-shell product **2** and mono-radical **3**. b) Molecular representation of homochiral hydrogen-bond-directed self-assembly of **2**, forming a honeycomb lattice (left), and **3**, forming a Kagome lattice (right).

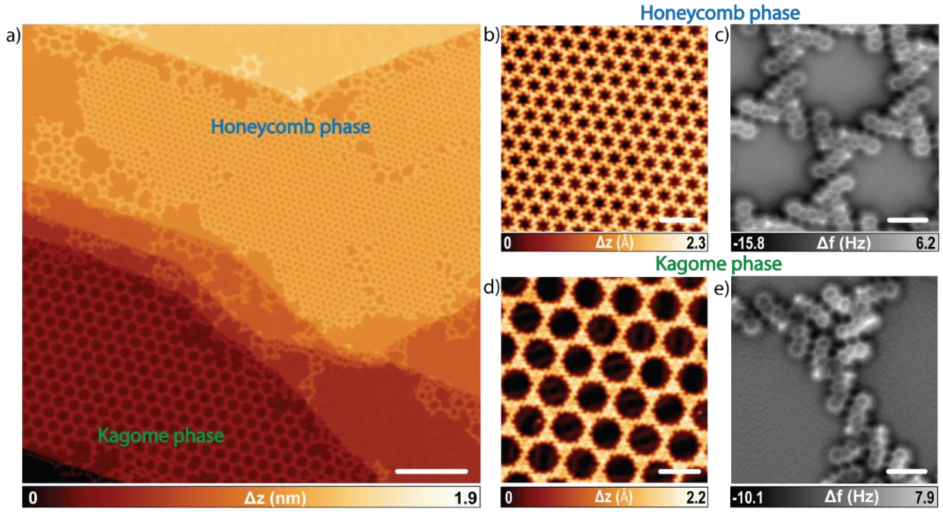

Figure S2. Hydrogen-bonded organic frameworks formed after annealing **1** on Au(111) at 325 °C. (a) Large-scale STM image showing the formation of the honeycomb and Kagome phases (V_b = 500 mV, I_t = 20 pA, scale bar = 40 nm). (b) Overview STM image of the honeycomb phase (V_b = 40 mV, I_t = 200 pA, scale bar = 7 nm). (c) Nc-AFM image of the honeycomb phase formed by product **2** (V_b = 1 mV, scale bar = 1 nm). (d) Overview STM image of the Kagome phase (V_b = 100 mV, I_t = 200 pA, scale bar = 7 nm). (e) Nc-AFM image of the Kagome phase formed by product **3** (V_b = 1 mV, scale bar = 1 nm).

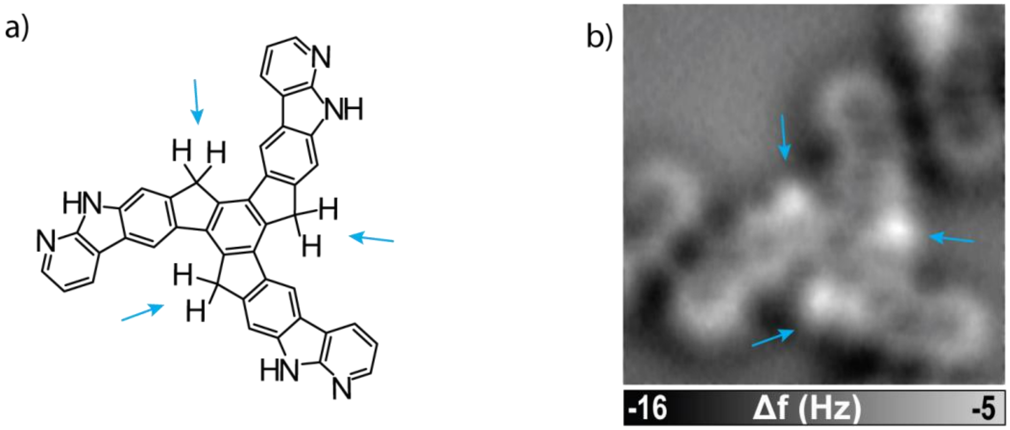

Figure S3. Structural characterization of **2** on Au(111). (a) Structure of **2**. Blue arrows highlight the apical carbon atoms of the five-membered rings with C-H bonds above and below the plane containing the conjugated molecule. (b) Nc-AFM image of **2** where the presence of three hydrogen atoms pointing out of the plane is elucidated through the bright spots (marked by blue arrows).

Scheme of the proposed reaction mechanism

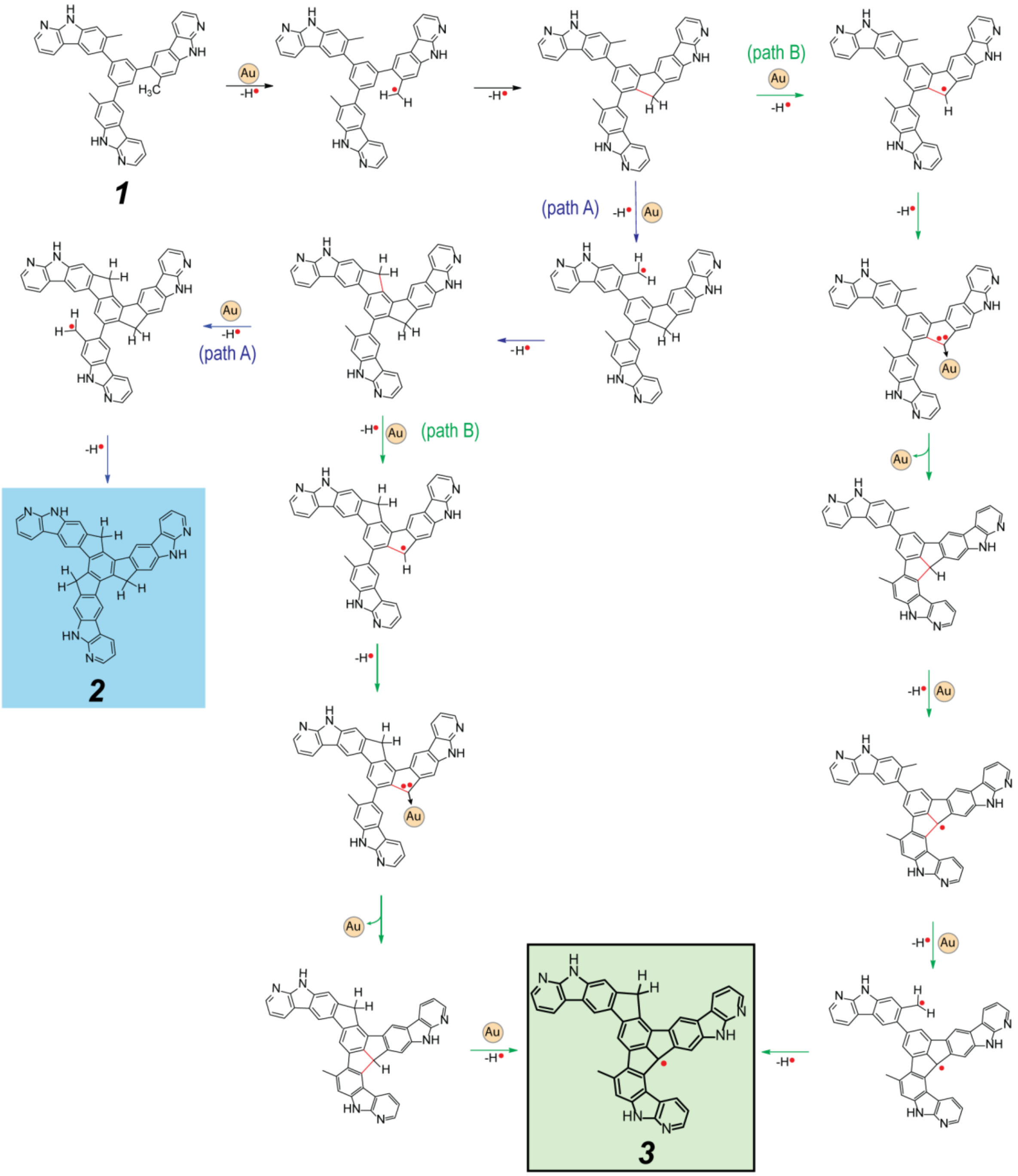

Scheme S3. Scheme of the reaction mechanism toward the formation of **2** and **3** on Au(111)

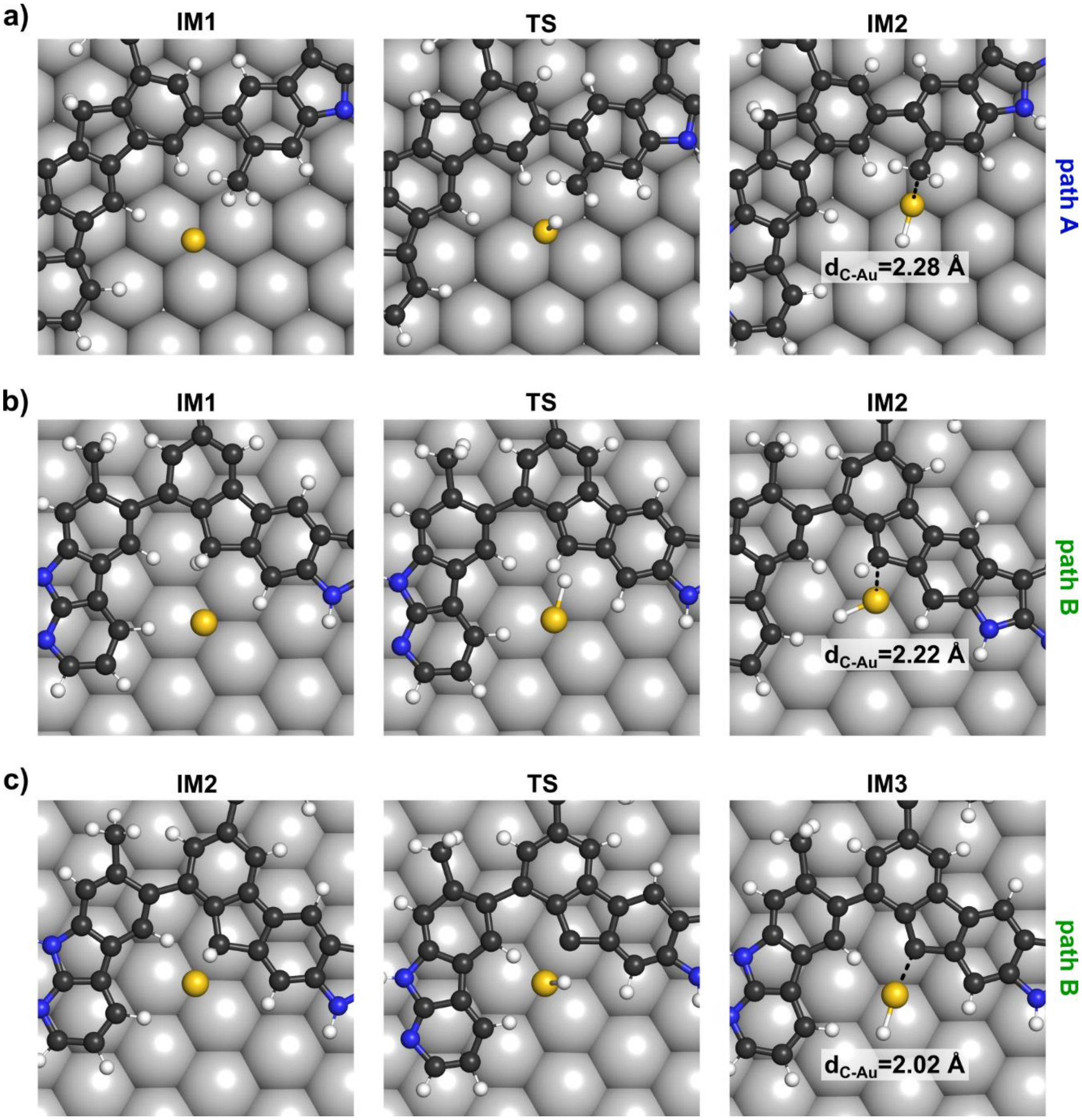

Figure S4. The atomic configuration of characteristic intermediate (IM) species involved in the dehydrogenation processes catalyzed by a single gold atom toward the formation of **2** (a) and **3**(b-c) obtained from free energy QM/MM simulations (see Figure 2 in the main text). The final configurations (IM2 and IM3) are energetically stabilized by the formation of Au-C bond, which passivates the radical character of the carbon involved in the cleavage of the C-H bond.

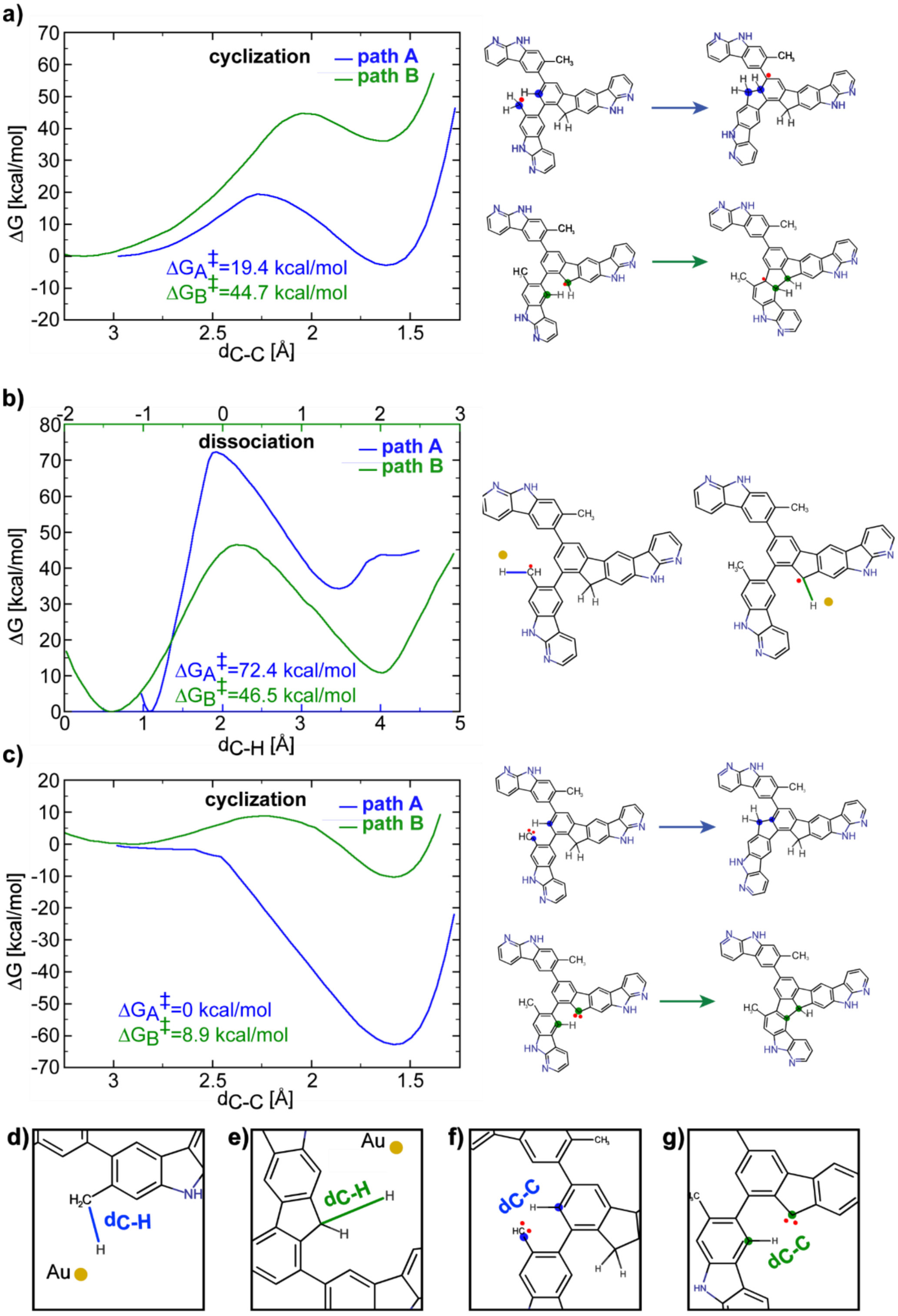

Figure S5. (a-c) Calculated free energy profiles for reaction steps following dissociations at bifurcations. (a) Cyclization step of formed mono-radicals. (b) Dissociation of mono-radicals to form carbenoid intermediates. (c) Cyclization of carbenoid species to form indenofluorene (path A), and fluoradene (path B). (d-g) Illustration of defined reaction coordinates used in presented umbrella sampling simulations.

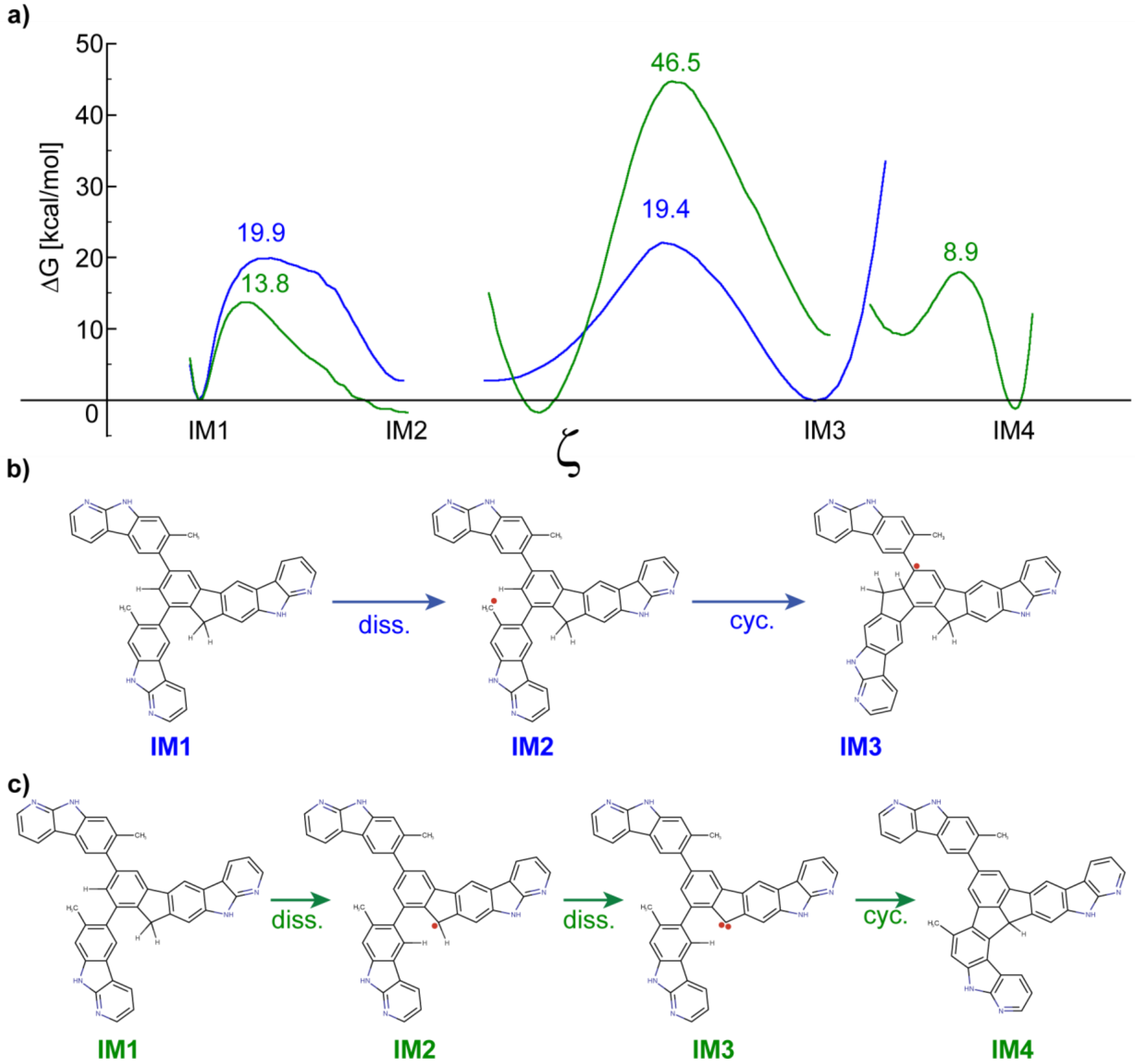

Figure S6. Proposed sequence of reaction steps at the first bifurcation. (a) Free energy profiles of consecutive steps aligned to the ΔG of the intermediate from the preceding step. Barrier heights are marked above each transition state. (b) Proposed sequence leading to the formation of indenofluorene after the first bifurcation. (c) Proposed sequence leading to the formation of fluoradene.

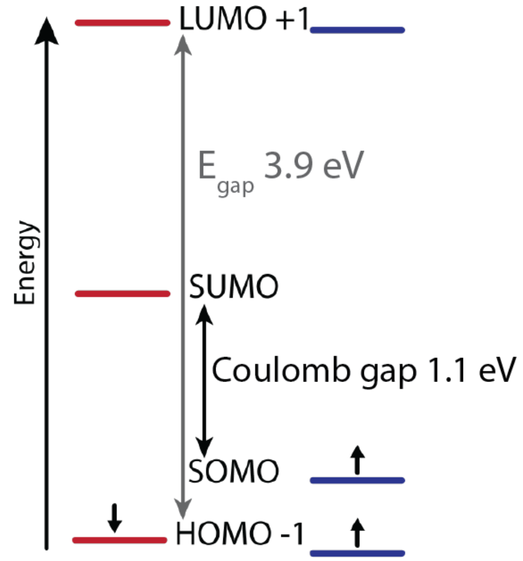

Figure S7. Schematic representation of the calculated spin diagram of **3.**

Table S1. Total energies of open-shell and closed-shell forms of **3**, a cluster of 4 Au atoms and 4 Au atoms with extra H atom, and the energy balance for different XC-functionals (PBE, PBE0, and B3LYP).

**Total energy/XC functionals**	**PBE (eV)**	**PBE0 (eV)**	**B3LYP (eV)**
Open-shell **3** (a)	-52861.9506	-52863.5536	-52925.3248
Closed-shell **3** (b)	-52878.4395	-52880.2215	-52942.0607
Au_4	-2142602.9384	-2142603.9492	-2142671.6703
Au_4H	-2142619.6239	-2142620.6365	-2142688.4789
H	-13.5980	-13.6278	-13.6565
H_2	-31.7373	-31.7980	-32.1144
$E_{3a}+Au_4H – E_{3b}- Au_4$	-0.1966	-0.0194	-0.0727
$E_{3a}+H_2 – E_{3b}- H$	-1.6504	-1.5023	-1.7220

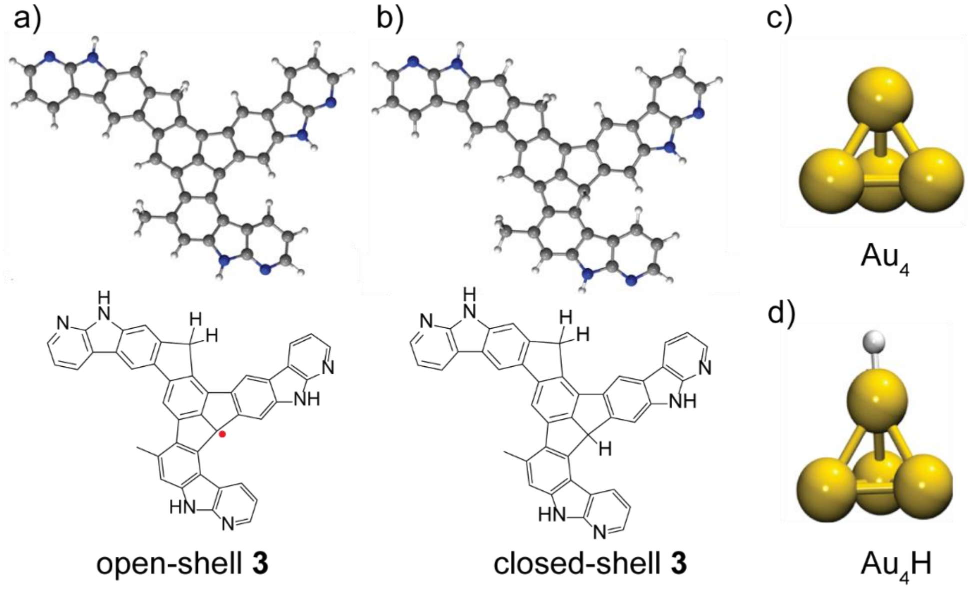

Figure S8. Analysis of thermodynamic stability of the open-shell (a) and the closed-shell (b) variant of product **3** in the presence of Au adatom (c). The closed-shell variant of **3** consists of passivation of fluoradene unit with extra hydrogen, as shown in Figure (b). A cluster of 4 Au atoms represents the gold adatom on Au(111) surface to make such calculations computationally feasible for hybrid exchange-correlation functionals. Figure (d) displays Au_4 with extra hydrogen atom.

References

(1) Giessibl, F. J. The qPlus Sensor, a Powerful Core for the Atomic Force Microscope. *Review of Scientific Instruments* **2019**, *90* (1), 011101. https://doi.org/10.1063/1.5052264.

(2) Gross, L.; Mohn, F.; Moll, N.; Liljeroth, P.; Meyer, G. The Chemical Structure of a Molecule Resolved by Atomic Force Microscopy. *Science* **2009**, *325* (5944), 1110–1114. https://doi.org/10.1126/science.1176210.

(3) Horcas, I.; Fernández, R.; Gómez-Rodríguez, J. M.; Colchero, J.; Gómez-Herrero, J.; Baro, A. M. WSXM: A Software for Scanning Probe Microscopy and a Tool for Nanotechnology. *Review of Scientific Instruments* **2007**, *78* (1), 013705. https://doi.org/10.1063/1.2432410.

(4) Case, D. A.; Aktulga, H. M.; Belfon, K.; Ben-Shalom, I. Y.; Brozell, S. R.; Cerutti, D. S.; T.E. Cheatham, I.; Cruzeiro, V. W. D.; Darden, T. A.; Duke, R. E.; Giambasu, G.; Gilson, M. K.; Gohlke, H.; Goetz, A. W.; Harris, R.; Izadi, S.; Izmailov, S. A.; Jin, C.; Kasavajhala, K.; Kaymak, M. C.; King, E.; Kovalenko, A.; Kurtzman, T.; Lee, T. S.; LeGrand, S.; Li, P.; Lin, C.; Liu, J.; Luchko, T.; Luo, R.; Machado, M.; Man, V.; Manathunga, M.; Merz, K. M.; Miao, Y.; Mikhailovskii, O.; Monard, G.; Nguyen, H.; O'Hearn, K. A.; Onufriev, A.; Pan, F.; Pantano, S.; Qi, R.; Rahnamoun, A.; Roe, D. R.; Roitberg, A.; Sagui, C.; Schott-Verdugo, S.; Shen, J.; Simmerling, C. L.; Skrynnikov, N. R.; Smith, J.; Swails, J.; Walker, R. C.; Wang, J.; Wei, H.; Wolf, R. M.; Wu, X.; Xue, Y.; York, D. M.; Zhao, S.; Kollman, P. A. Amber20. **2021**.

(5) Lewis, J. P.; Jelínek, P.; Ortega, J.; Demkov, A. A.; Trabada, D. G.; Haycock, B.; Wang, H.; Adams, G.; Tomfohr, J. K.; Abad, E.; Wang, H.; Drabold, D. A. Advances and Applications in the F IREBALL Ab Initio Tight-Binding Molecular-Dynamics Formalism. *physica status solidi (b)* **2011**, *248* (9), 1989–2007. https://doi.org/10.1002/pssb.201147259.

(6) Heinz, H.; Lin, T.-J.; Kishore Mishra, R.; Emami, F. S. Thermodynamically Consistent Force Fields for the Assembly of Inorganic, Organic, and Biological Nanostructures: The INTERFACE Force Field. *Langmuir* **2013**, *29* (6), 1754–1765. https://doi.org/10.1021/la3038846.

(7) Kumar, S.; Rosenberg, J. M.; Bouzida, D.; Swendsen, R. H.; Kollman, P. A. THE Weighted Histogram Analysis Method for Free-Energy Calculations on Biomolecules. I. The Method. *Journal of Computational Chemistry* **1992**, *13* (8), 1011–1021. https://doi.org/10.1002/jcc.540130812.

(8) Grossfield, A. WHAM: The Weighted Histogram Analysis Method, Version 2.0.9. http://membrane.urmc.rochester.edu/wordpress/?page_id=126.
